# Metabolic skinflint or spendthrift? Insights into ground sloth integument and thermophysiology revealed by biophysical modeling and clumped isotope paleothermometry

**DOI:** 10.1007/s10914-024-09743-2

**Published:** 2025-01-14

**Authors:** Michael D. Deak, Warren P. Porter, Paul D. Mathewson, David M. Lovelace, Randon J. Flores, Aradhna K. Tripati, Robert A. Eagle, Darin M. Schwartz, Michael T. Butcher

**Affiliations:** 1 Department of Chemical and Biological Sciences, Youngstown State University, Youngstown, OH USA; 2 Department of Integrative Biology, University of Wisconsin - Madison, Madison, WI USA; 3 Department of Geosciences, University of Wisconsin - Madison, Madison, WI USA; 4 Department of Earth and Space Sciences, University of California - Los Angeles, Los Angeles, CA USA; 5 Department of Atmospheric and Oceanic Sciences, Institute of the Environment and Sustainability, Center for Diverse Leadership in Science, University of California - Los Angeles, Los Angeles, CA USA; 6 Department of Geosciences, Boise State University, Boisie, ID USA

**Keywords:** Body temperature, Fur, Geochemistry, Metabolism, Paleoclimate, Paleontology, Skin, Xenarthra

## Abstract

Remains of megatheres have been known since the 18th -century and were among the first megafaunal vertebrates to be studied. While several examples of preserved integument show a thick coverage of fur for smaller ground sloths living in cold climates such as *Mylodon* and *Nothrotheriops*, comparatively very little is known about megathere skin. Assuming a typical placental mammal metabolism, it was previously hypothesized that megatheres would have had little-to-no fur as they achieved giant body sizes. Here the “hairless model of integument” is tested using geochemical analyses to estimate body temperature to generate novel models of ground sloth metabolism, fur coverage, and paleoclimate with Niche Mapper software. The simulations assuming metabolic activity akin to those of modern xenarthrans suggest that sparse fur coverage would have resulted in cold stress across most latitudinal ranges inhabited by extinct ground sloths. Specifically, *Eremotherium* predominantly required dense 10 mm fur with implications for seasonal changes of coat depth in northernmost latitudes and sparse fur in the tropics; *Megatherium* required dense 30 mm fur year-round in its exclusive range of cooler, drier climates; *Mylodon* and *Nothrotheriops* required dense 10–50 mm fur to avoid thermal stress, matching the integument remains of both genera, and further implying the use of behavioral thermoregulation. Moreover, clumped isotope paleothermometry data from the preserved teeth of four genera of ground sloth yielded reconstructed body temperatures lower than those previously reported for large terrestrial mammals (23 ± 5–32 ± 3° C). This combination of low metabolisms and thick fur allowed ground sloths to inhabit various environments.

**Supplementary information:**

The online version contains supplementary material available at 10.1007/s10914-024-09743-2.

## Introduction

Xenarthrans have a rich and diverse history preserved in the fossil record dating back 60 million years ago (MYA) to the Eocene Epoch. The Xenarthra consists of the clades Cingulata, comprising living armadillos and the extinct pampatheres and glyptodonts, and Pilosa, which contains anteaters and sloths. While the genera *Choloepus* (two-toed sloths) and *Bradypus* (three-toed sloths) are the only extant linages of (tree) sloths, numerous extinct genera of sloths (i.e., arboreal, terrestrial, and aquatic) once occupied various niches during the Oligocene and persisted until the Late Pleistocene. Ground sloths thrived throughout South America and dispersed north to eventually colonize North America during the late Cenozoic Great American Biotic Interchange (GABI) (Feldhamer et al. 2007). Several extinct ground sloth taxa such as *Eremotherium* and *Megatherium* became the largest xenarthrans ever in existence, reaching sizes comparable to or exceeding that of modern African savanna elephants.

To date, preserved integument that is definitively attributed to the largest ground sloths *Eremotherium* and *Megatherium* has yet to be found. The only specimen of *Megatherium* having a sample of skin was lost before it could be inventoried (Pujos and Salas 2004). Nonetheless, both taxa have been historically reconstructed as having a thick coat of long, shaggy fur similar to that of smaller ground sloths (e.g., *Mylodon* and *Nothrotheriops*) and extant tree sloths. This appearance, however, was challenged on the basis of thermal energetics suggesting a largely hairless integument in the largest ground sloths (McNab 1985; Fariña 2002). These analyses utilized the following thermal conductivity equation: (Φ_cond_ = (k_skin_/s) × (T_inner_– T_skin_), where Φ_cond_ is the heat flow density of the skin, k_skin_ is the thermal conductivity of the skin, s is the skin thickness, and core body temperature and ambient temperature is represented by T_inner_ and T_skin_, respectively. Fariña (2002) hypothesized that given their massive sizes, the large-bodied ground sloths would not have been thermally neutral if they were covered in thick fur. However, if the integument of *Megatherium* closely mirrored that of the largest extant terrestrial mammals (i.e., elephants, hippopotamuses, and rhinoceroses), then it would be in a thermoneutral zone even if the surrounding temperatures were nearly − 17° C on average. A thermoneutral zone is defined as a range of ambient temperatures where an endotherm can remain in heat balance without metabolic heat production or requiring evaporative heat loss. While it is tempting to make comparisons to extensively furry megafauna such as the woolly mammoth, it should be noted that these comparisons are not applicable to megatheres. Woolly mammoths had a suite of adaptations for living in the frigid Siberia tundra such as reduced ear and tail sizes and an insulating layer of fat (Boeskorov et al. 2016), as well as hemoglobin that prevented their blood from freezing (Campbell et al. 2010).

The “hairless model of integument” was again recently evoked by Lindsey et al. (2020) regarding a mass assemblage of *Eremotherium* remains recovered from Tanque Loma in Ecuador. The cause of death of these animals was believed to be disease (or drought) from fecal matter contamination of the marsh. Lindsey et al. (2020) postulated that wallowing behavior would be advantageous if *E. laurillardi* had a hairless integument, as it would offer protection against the sun and tropical insects, and thus would be similar to a protective strategy used by the largest-bodied extant terrestrial mammals.

Despite evidence in favor of a hairless (or nearly hairless) model of integument in the largest ground sloths that ever lived, there were critical assumptions applied to the previous methodologies that may not be tenable. First, the thermal conductivity equation used by Fariña (2002) only accounts for core body temperatures and the effects of ambient temperatures alone on thermoneutrality without accounting for other environmental factors. While this calculation is useful in determining heat conduction flux, other environmental factors that could impact thermoneutrality such as relative humidity and wind speed, in addition to variations in ambient temperature across the broad geographic distribution of the ground sloth genera should have been considered. Second, the previous model (Fariña 2002) did not accurately account for the basal metabolism of extant xenarthrans for calculations of heat flux, or the thick skin of living sloths that would affect heat loss. Instead, the basal metabolic rate of a naked human was isometrically scaled up to the size of *Megatherium* assuming similar core body temperature and skin thickness to that of hominids (Fariña 2002). Third, determinations of the basal metabolic rate of *Megatherium americanum* were made by halving the estimated metabolic rate, thus leading to the conclusions that it could still be furless and that temperatures as low as -10° C would be within its thermoneutral zone. Fourth, thermoneutral zone estimates derived from the previous model for smaller ground sloth taxa are inconsistent with known paleoclimate data and preserved integument. Temperatures of -4° C and − 28° C, respectively, have also been calculated as being within the thermoneutral zone for a completely furless *Mylodon darwinii* versus a conspecific with a fur coat 4 cm long, but with each having an equivalent mass of 2,000 kg. (Fariña 2002).

The fossil evidence known for *Mylodon*, however, indicates that it was extensively covered in fur, which was as long as 6.5–10 cm in some regions of its body (Collins 1933; Santos et al. 2024). Stable isotopes found in contemporary samples of *Notiomastodon* enamel dating to 33,661–10,239 years ago have also produced mean annual temperature estimates ranging between 9º C and 21º C (González-Guarda et al. 2017). Moreover, the relationship between body mass and body temperature in mammals is complex and purported to be mediated primarily through ecology (Clarke and Rothery 2008). Recent data on large extant mammal energetics indicate that the thermoneutral zone only accounts for favorable ambient temperature ranges where little energy needs to be expended beyond the basal metabolic rate, and not the full extent of the normal functions associated with daily activity such as foraging. Thus, prescriptive zones, tolerance zones, and survival zones have been adopted by other authors (Mitchell et al. 2018; Boyles et al. 2019; Levesque and Marshall 2021), which encompass temperatures where an endotherm can function normally, temperatures where thermal stress occurs, and temperatures where death by thermal stress occurs, respectively.

Recently, Varela et al. (2024) attempted to estimate the metabolic rates of several extinct xenarthrans by measuring the diameter of the femoral nutrient foramina. Estimates of blood flow obtained from an equation developed by Seymour et al. (2019) resulted in metabolic rates in fossil xenarthrans comparable to non-xenarthran placental mammals of similar size. While these findings lend support to speculation that ground sloths possessed a typical placental metabolism due to increased skeletal muscle mass compared to living tree sloths (Fariña 2002), and faced environmental conditions below proposed thermal neutrality (Tejada et al. 2021), other data suggests a more xenarthran-like metabolism. For example, estimates of the body temperature in *Nothrotheriops* (based on amino acids preserved in bone collagen) showed that it had a range of body temperature values akin to extant xenarthrans (Ho 1967). Similar estimates of the metabolic rates and thermal conductivity for both *Mylodon* and *Nothrotheriops* suggest that each had low basal metabolic rate and relatively poor insulation compared with other mammals of similar size (McNab 1985). Ground sloths plausibly may have had lower metabolic rates than other mammals of their size, although this tentative conclusion does not imply that they were as slow-moving as living tree sloths. Using step length from a fossilized trackway at Pehuén Có, Argentina attributed to *Megatherium*, Blanco and Czerwonogora (2003) predicted that its average walking speed was ~ 1.21 ms^− 1^, which is 10 times faster than extant *Bradypus* suspensory walking below-branch (Gorvet et al. 2020; McKamy et al. 2023). A maximum speed of nearly 2.2 ms^− 1^, which is still considerably slower than the top velocity of 5–6 ms^− 1^ measured for elephants (Hutchinson et al. 2006), also has been estimated for *Megatherium* (Billet et al. 2013). Despite ground sloths, elephants, and tree sloths having different modes of locomotion, their reported speeds emphasize the general principle that animals with low metabolic rates tend to move more slowly than those with higher metabolic rates.

The objective of this research is to further test the hairless model of integument for the largest ground sloths, *Eremotherium* and *Megatherium*, and to place novel constraints on their thermoregulation. To rigorously test the model through quantitative means, numerous climatic and physiological factors were input, for example core body temperature, metabolic rate, and estimates of paleoclimate. Simultaneously, integrating these variables will provide more insight into the reconstruction of large ground sloth integument until skin and/or fur samples are discovered. In addition to physiological and ecological constraints on integument, a better understanding of the evolution of core body temperature and/or metabolic rate across various extinct and extant phylogenetic branches of the Folivora is an expected outcome of this study.

## Materials and methods

### Fossil specimens and sample preparation

Tooth samples from *Eremotherium* (*N* = 2), *Megatherium* (*N* = 1), *Promegatherium* (*N* = 1), and *Nothrotheriops* (*N* = 1) were used in a series of geochemical analyses (Table 1). Cortical bone samples from either the same specimen or from another in the same general locality were also used to determine the degree of diagenesis via an analysis of rare earth element (REE) uptake in both cortical bone and tooth samples. The specimens obtained were from the collections of the Field Museum of Natural History in Chicago, IL (FMNH) and the University of Florida in Gainesville, FL (UF). No permits were required for the present study, which complied with all relevant legal regulations. While no samples of *Mylodon* were obtained for this study, body temperature estimates for this genus were extrapolated from values obtained from *Promegatherium* given that both genera were similar in body size. This comparison based on body size rather than phylogeny is justifiable given that *Bradypus* and *Choloepus* are similar in size and metabolic capabilities despite being distantly related (McNab 1985; Cliffe et al. 2023). The sampling strategy accounted for a wide collective geographic distribution of these genera from the southernmost extreme of their range in Argentina to the northernmost extreme of their range in Florida.

**Table 1:** Body temperature estimates from clumped isotopic analyses and diagenesis ratios from rare earth element (REE) analyses. *Abbreviations*: **SD**, standard deviation; **SE**, standard error

Genus/ Sample ID	Locality	Depositional environment	*N* ^a^	REE^b^	δ^13^C (‰, V-PDB) ± SD	δ^18^O (‰V-PDB) ± SD	Δ_47_ (‰) ± SE	T_Δ47_ (°C)^c^ ± SE	δ^18^O_w_ (‰, V-SMOW)^d^ ± SD
*Eremotherium* UF 95869	Inglis 1 A, Citrus County, Florida, USA	Sinkhole	5	0.42	-9.01 ± 0.02	-1.11 ± 0.08	0.582 ± 0.009	29 ± 2	2.5 ± 1.7
*Nothrotheriops* UF 87131	Leisey 1 A, Hillsborough County, Florida, USA	Estuary	4	0.86	-9.48 ± 0.26	0.90 ± 0.29	0.573 ± 0.008	32 ± 3	5.4 ± 1.3
*Megatherium* FMNH P13725	Tarija Valley, Argentina	Fluvial	4	0.12	-3.65 ± 0.04	-4.39 ± 0.19	0.577 ± 0.003	31 ± 1	-0.3 ± 0.7
*Eremotherium* UF 312730	Halie 7G, Alachua County, Florida, USA	Lacustrine/ karst	4	0.02	-4.28 ± 0.27	-1.65 ± 1.64	0.694 ± 0.031	-3 ± 7	-8.1 ± 6.6
*Promegatherium* FMNH 14404	Puerta de Corral Quemado, Argentina	Terrestrial indet.	3	36.6	-6.49 ± 0.05	-3.39 ± 0.19	0.602 ± 0.014	23 ± 5	-1.7 ± 2.4

^a^ Number of replica stable isotope measurements from the same sample

^b^ Rare earth element ratio between dentin and cortical bone (see Methods)

^c^ Δ_47_ temperature (Anderson et al. 2021)

^d^ δ^18^Ow (where “w” signifies mineral formation waters; Lécuyer et al. 2010)

**Fig. 1:**
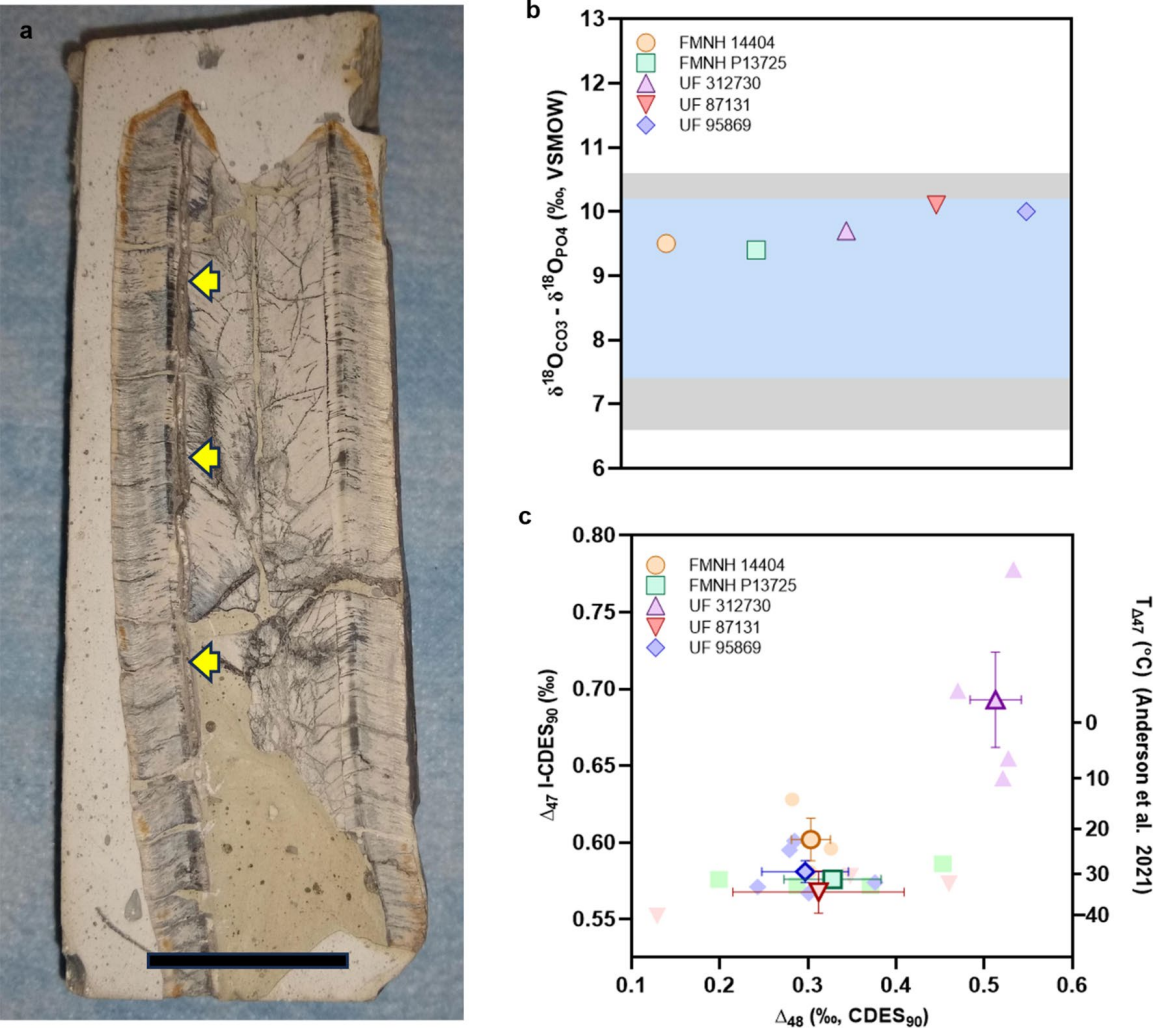
**a.** The tooth of *Megatherium* FMNH P13725 with sampled regions indicated by yellow arrows; scale bar equals 30 mm. **b.** Percentage of phosphate δ^18^O in the sampled ground sloth teeth compared to the ranges reported for extant placental mammal bioapatite; blue and gray shaded areas indicate 95th and 99th percentile, respectively; mammal bone and enamel data from Eagle et al. (2011). **c**. Scatter plot of the relationships between ∆47, ∆48, and estimated temperatures of the sampled teeth; symbols with light outlines represent individual samples, and symbols with bold outlines and error bars represent the mean and standard deviations of the samples

Given that the outer dentin of fossil xenarthran teeth are susceptible to large quantities of rare earth element uptake (MacFadden et al. 2010), inner orthodentine was preferentially sampled, as a recent study (Larmon et al. 2019) using cathodoluminescence revealed that this dental material is the most resistant tissue to diagenesis and exhibits the least amount of mineral impregnation compared to other dentine layers. Approximately 300–400 mg of powderized internal orthodentine (and cortical bone) were harvested with a power hand drill fit with a 0.7 mm diamond-tipped drill bit. Drilled samples of inner orthodentine were taken from several random sites along the entire length of the tooth starting at the apex to the root (Fig. 1a). To remove any organic contaminants, the samples were washed with 5.0 ± 0.5 ml of 3% H_2_O_2_ for 3 h in 15 ml conicals. The tubes remained open so that CO_2_ could be released during purification. The samples were then pelleted in a centrifuge at 10k rpm (centrifugation radius = 12 cm; centrifugal force = 13,416 g) for 10 min. Specifically, all samples were centrifuged at a constant temperature of 25º C, followed by 3 rinses in room temperature ddH_2_O water. Any remaining liquid was decanted before drying overnight in an oven at 50º C. Extra drying time in the oven was necessary for some of the samples to ensure complete desiccation of contaminants so that the samples were dried to a concentrated mass.

### Geochemical alteration analysis

To assess the amount of diagenesis present in the samples, REE analyses were performed on subsamples (5–10 mg) of prepared inner orthodentine and cortical bone (MacFadden et al. 2010). For analysis, samples were weighed by microbalance and digested in a mixture of 0.1 mL 29 M HF and 0.3 mL 16 M HNO_3_ on a hotplate at 150 °C. Samples were dried down and redissolved in 0.5 ml of 16 M HNO_3_ three times prior to diluting gravimetrically to 2% HNO_3_ for analysis. Samples were prepared along-side a suite of geologic and bone standard powders and total procedural blank following the same dissolution procedure, for use as calibration and external reference materials. Analyses were performed on a Thermo Scientific, iCAP-RQ, single quadrupole mass spectrometer using a PFA micromist nebulizer; peltier-cooled quartz, cyclonic spray chamber, 2.5 mm quartz injector and quartz torch; Ni cones, high matrix (3.5 mm) skimmer insert. Samples were uptaken using an ESI SC 4DX FAST autosampler using the FAST 2.0 mL method, with a stabilization time of 30s and dual rinse (4 s each) in 4% HNO3 + trace HF following each analysis. Samples were mixed in real-time with a 2 ppb In solution using the FAST autosampler, which was used as an internal standard for the experiment. Reported analyses are the mean of 5 measurements, which consist of 25 sweeps on ^44^Ca, ^45^Sc, ^88^Sr, ^89^Y, ^115^In, ^137^Ba, ^139^La, ^140^Ce, ^141^Pr, ^146^Nd, ^147^Sm, ^153^Eu, ^157^Gd, ^159^Tb, ^163^Dy, ^165^Ho, ^166^Er, ^169^Tm, ^172^Yb, ^175^Lu, ^208^Pb, ^238^U, for 10 ms each. Raw counts were normalized to a uniform intensity over the run by normalizing against In. Concentrations were converted from normalized counts using Thermo Scientific Qtegra software (eQuant) by linear regression of the calibration standards (BHVO-2, BCR-2, BIR-1, AGV-2; Jochum et al. 2016), anchored to the total procedural blank. Accuracy and precision were monitored using whole rock powder W2 (Jochum et al. 2016), NIST1400 bone ash, NIST1486 bone meal and NOCS fossil bone material (Chavagnac et al. 2007) and multiple dilutions of a mixed 100 ppm REE standard solution (BDH82025-926) diluted to similar concentrations as the unknowns.

All measurements were normalized against Post-Archean Australian Shale (PAAS; Nance and Taylor 1976) to compensate for the effects of the Oddo-Harkins even-odd abundance effect. While MacFadden et al. (2010) reported a significant increase in REE uptake in fossils compared to modern bone and dental tissues, an REE index was established to assess the degree of diagenesis in fossil xenarthran teeth compared to enamel samples from similar localities. The sum of all normalized REE (REE_N_) present in orthodentine were divided by the sum of all normalized REE found in cortical bone and expressed as a ratio (REE_N_ sample/REE_N_ cortical bone). Ratios > 1.0 indicate significant geochemical alteration signifying that isotopic geochemical data should be treated with caution, whereas sample ratios between 0.5 and 1.0 indicate mild to moderately altered samples, and ratios > 0.35 (akin to most samples of fossil enamel from non-xenarthrans) indicates a sample with little to no geochemical alteration (MacFadden et al. 2010).

Samples were prepared for phosphate δ^18^O analysis following the rapid silver phosphate precipitation protocol outlined in Mine et al. (2017) and detailed in Conte et al. (2024). In brief, approximately 1.0 mg of sample material was weighed out in 2 mL microcentrifuge tubes and dissolved overnight in 2.0 M nitric acid (HNO_3_). Calcium fluoride (CaF_2_) was then precipitated with the addition of 2.9 M hydrofluoric acid (HF) and 2.0 M sodium hydroxide (NaOH). Samples were centrifuged to pellet CaF_2_ and the phosphate ion containing supernatant was transferred to a new clean microcentrifuge tube. The CaF_2_ pellet was rinsed with 0.1 M sodium fluoride (NaF) to recover residual phosphate, and the resulting supernatant aliquot was combined with the first aliquot. Sample phosphate was precipitated as silver phosphate (Ag_3_PO_4_) with a silver amine solution containing 1.09 M ammonium hydroxide (NH_4_OH) and 0.37 M silver nitrate (AgNO_3_) at a pH range of 5.5 to 7.5. Adjustments to pH were made with the addition of small aliquots of 2 M HNO_3_ or 2 M NaOH and samples were allowed to react for 10 min. Samples were centrifuged to pellet Ag_3_PO_4_ crystals and following the removal of supernatant fluid were rinsed five times with distilled water and dried overnight in a 50° C oven. Dried samples were weighed out to ~ 150–200 µg in packed silver capsules and analyzed in triplicate using a ThermoFisher Thermal Conversion Elemental Analyzer (TC/EA) paired with a Delta V plus Continuous Flow Isotope Ratio Mass Spectrometer in the Stable Isotope Ecosystem Laboratory of UC Merced (SIELO). Reference material NIST SRM 120 C was precipitated and analyzed alongside sample unknowns as a preparation standard and yielded a δ^18^O value of 21.7 ± 0.05 (*N* = 3). Reference materials USGS 80 (δ^18^O = 13.1 ± 0.3; *N* = 15) and USGS 81 (δ^18^O = 35.4 ± 0.3; *N* = 15) were used for linearity and drift corrections and standardization.

### Bioapatite stable isotope geochemistry and body temperature estimates

Carbonate clumped isotope thermometry is an approach to determine mineral formation temperatures based on measurements (which are reported as Δ_47_ values) of the abundance of ^13^C-^18^O bonds in carbonate moieties (Ghosh et al. 2006). The Δ_47_ values found in tooth bioapatite reflect the body temperature of the organism that formed them (Eagle et al. 2010). Furthermore, Δ_47_ measurements of vertebrate biominerals are able to distinguish between endothermic and ectothermic physiology (Eagle et al. 2011, 2015; Griffiths et al. 2023), as well as between endotherms with different metabolic rates. For example, these techniques have played a crucial role in resolving body temperature differences between regionally endothermic sharks, large terrestrial and marine mammals, and between mammals and birds. Measurements from fossils also yielded plausible body temperatures for extinct vertebrates across of a range of epochs (Eagle et al. 2010, 2011, 2015; Griffiths et al. 2023).

All samples were analyzed via stable isotope ratio mass spectrometry. Specifically, samples of ground sloth inner orthodentine were digested in a common acid bath containing 105 wt% phosphoric acid reacted at 90° C followed by multiple steps of cryogenic purification and passage through either a gas chromatograph column (MAT 253 mass spectrometer, Thermo Fisher Scientific, USA) or adsorption trap (Nu Perspective mass spectrometer, Nu Instruments-AMETEK, UK) packed with Porapak Type-QTM 50/80. Following sample purification, CO_2_ gas was then introduced into the isotope ratio mass spectrometer (IRMS) systems via an automated changeover block, thus allowing for continuous alternating measurements of sample and reference CO_2_ gas on detectors configured to measure isotopologues of masses 44 to 49. Measurements performed on the Thermo MAT 253 mass spectrometer (~ 60–80 mg of sample material) consisted of nine blocks of 10 cycles totaling 720 s of integration time at a signal of 16–15.7 V over the course of a block with pressure adjustments between each block to return to a beam of 16 V at the start of each new block. Measurements taken with the Nu Perspective mass spectrometer (~ 7–10 mg of sample material) consisted of three blocks of 20 cycles totaling in 1,200 s of integration at a signal which ranged from 80 to 30 nA on mass 44 over the course of each acquisition. Carbonate standards ETH-1 and ETH-2 were used to perform a nonlinearity correction on sample unknowns. These nonlinearity corrected values were then projected into an I-CDES reference frame (Bernasconi et al. 2021) using ETH-1-2, and ETH-3 along with three in-house standards. ETH-4 was not included in the corrections and was instead used as a check for data quality. Corrections were applied to tooth samples and standard data over a moving average of 10 standards on either side of a given sample and were calculated using Easotope software (John and Bowen 2016). Reconstructed temperatures were derived from a Δ_47_-temperature calibration method (Anderson et al. 2021) that has recently demonstrated that using the latest I-CDES reference frame a bioapatite calibration was statistically indistinguishable from the calibration used in these analyses (Anderson et al. 2021; Griffiths et al. 2023). Equations used to estimate the formation of waters during mineralization are published elsewhere (Lécuyer et al. 2010).

An accepted limitation in the application of clumped isotope thermometry for assessing vertebrate physiology is the preservation of original isotope signatures and the possibility of post-depositional diagenesis, and how these factor influence the accuracy of the estimates. While precisely constraining diagenesis can be complex, the following two approaches were employed: (1) determining the REE concentration of fossil ground sloth teeth and (2) comparing measurements of δ^18^O of both the carbonate and phosphate moieties of fossil bioapatite. As the carbonate moiety may be more susceptible to chemical alteration than the phosphate moiety deviation, the offset between the range of carbonate and phosphate seen in extant organisms has been used to indicate preferential diagenesis of the carbonate moiety (Eagle et al. 2011). Rare earth elements can be taken up by bioapatite during the fossilization process and very high REE content may be indicative of recrystallization of the fossil apatite (McFadden et al. 2010).

### Modeling of animals and paleoclimate

**Fig. 2:**
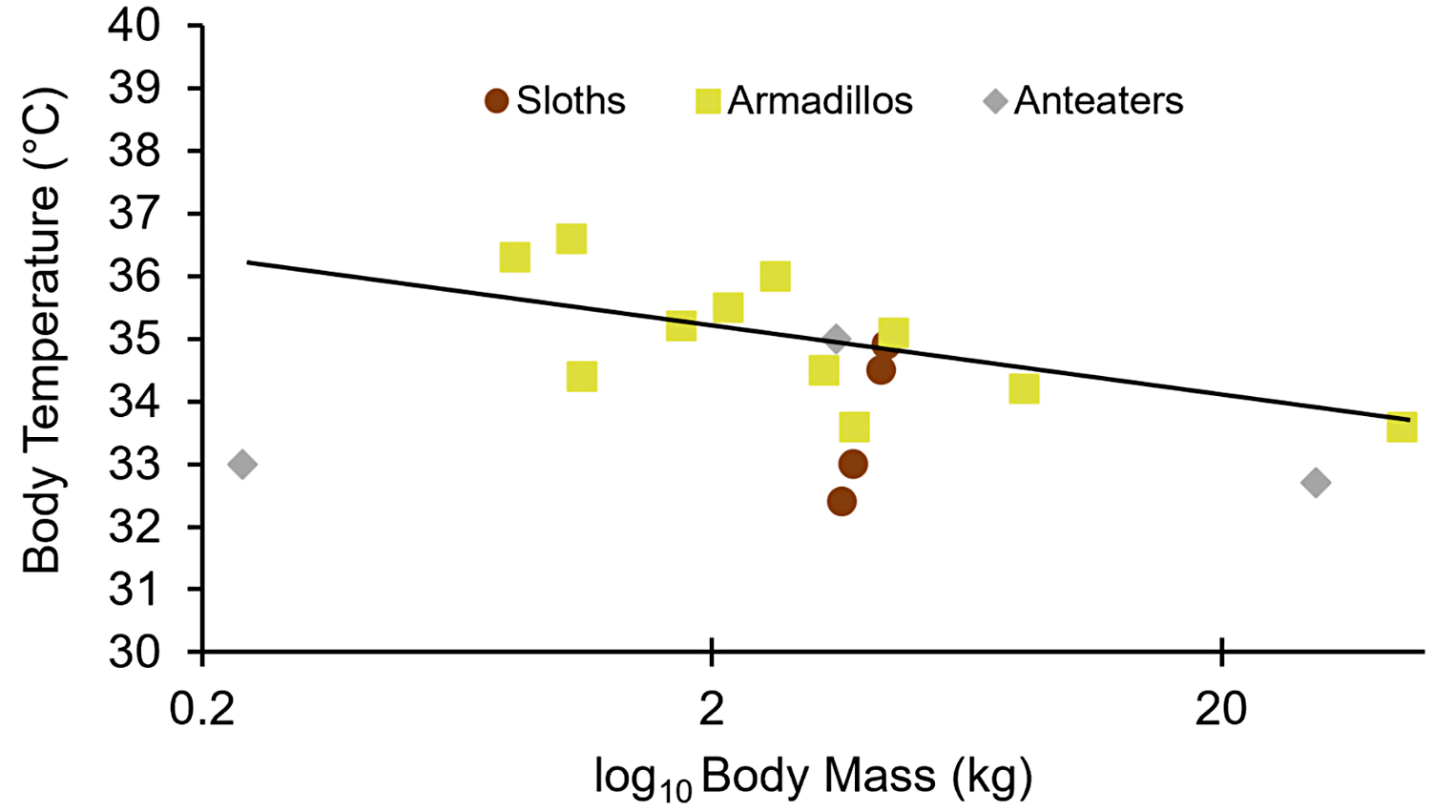
Regression of extant xenarthran body mass against body temperature (y = 34.908x^− 0.01^; R^2^ = 0.1833). The data include three sloth species (*N* = 4), eight armadillo species (*N* = 11), and three anteater species (*N* = 3) and were obtained from the literature (McNab 1985; Cliffe et al. 2018; Seitz and Puig 2018)

Body temperature estimates obtained from the literature for extant xenarthrans (McNab 1985; Cliffe et al. 2018; Seitz and Puig 2018) were evaluated via linear regression to determine the relationship between core body temperature and body size for all initial simulations. Temperature (in ºC) was log-transformed and plotted against the log of mass (in kg) to establish an expected relationship. These data showed no relationship among extant xenarthrans (Fig. 2); however, extant xenarthran body masses range between 0.2 and 45.2 kg, thus none of the taxa used for this scaling relationship approximate the body size of extinct ground sloths. Nonetheless, modeling simulations were meant to represent an average adult-sized animal at any given point in geologic time, but are not meant to be based on a specific specimen such as those used in the clumped isotope analyses. Body temperature estimates, derived from clumped isotope paleothermometer analyses among various other environmental and physiological variables were input into the broadly tested biophysiological modeling software, Niche Mapper. The microclimate sub-model of Niche Mapper converts inputs of macroclimate values into the warmest (minimum shade) and coolest (maximum shade) microclimates simultaneously from 2 m above ground to 2 m below ground. The animal sub-model then uses the hourly results in order to find a microenvironment suitable for optimal thermal comfort, while allowing the user to define various morphological, behavioral, and physiological inputs, including animal body geometry, user defined metabolic rate, denning and burrowing behaviors, etc. Both sub-models solve for heat and mass balances of the local substrates and the animal, respectively, in addition to finding the best available microclimate for each hour of a simulated day to keep the simulated animal within the closest possible range of the thermoneutral zone.

Recent work with Niche Mapper has been used to predict both environmental and physiological constraints on various extinct taxa, including mammoths (Wang et al. 2018, 2020) and Triassic dinosaurs (Lovelace et al. 2020). Basal metabolic rate was estimated using a published metabolic scaling relationship derived from 15 extant xenarthran species (3.14 M^0.69^;White and Seymour 2004), where M is body mass in grams, and the resulting basal metabolic rate in ml O_2_/hr^− 1^ was converted into the unit of watts. An activity multiplier of 2 was used to estimate values of field metabolic rate. The scaling relationship (3.3 M^0.76^; Fariña 2002), where M is body mass in kg, was originally derived from Kleiber (1932) and it was used to compare metabolic rates derived from a mammal of similar body size with the typical placental metabolism.

Three-dimensional computer models for both *Eremotherium* and *Mylodon* were fitted with non-uniform rational basis spline (NURBS) curves and used to estimate body volumes, surface areas, and rate of heat dissipation, and these data further served as proxies for the same computed parameters in metabolic models for *Megatherium* and *Nothrotheriops*, respectively. The models were constructed in ZBrush by paleoartists, and we’re used in this study with permission. While distantly related phylogenetically, the 3D model of *Mylodon* was specifically used as a proxy for *Nothrotheriops* given that both taxa were more similar in body size than either were to the megatheres. Mass estimates for *Eremotherium* (4,490 kg; Barbosa et al. 2023), *Megatherium* (3,706 kg; Brassey and Gardiner 2015), *Mylodon* (1,986 kg; Christiansen and Fariña 2003), and *Nothrotheriops* (463 kg; McDonald 2005) were input into these simulations. While higher mass estimates have been reported in the literature for both *Eremotherium* and *Megatherium* (6,550 kg; Dantas et al. 2017; 6,000 kg; Fariña et al. 1998), these were treated as high-end estimates herein, and more conservative body masses were used instead. Models of full body fur coverage were tested at coat thicknesses of 10, 30, and 50 mm for all four taxa. Fur densities that were tested specifically consisted of “dense” fur of 2,000 hairs/cm^2^ (Lovelace et al. 2020) and 1,300 hairs/cm^2^ (e.g., horse flank fur density; Tregear 1965), in addition to “sparse” fur of 8.5 hairs/cm^2^ (e.g., pig flank fur density; Tregear 1965) and 0.07 hairs/cm^2^ (maximum elephant fur density; Myhrvold et al. 2012).

Metabolic chamber simulations for each taxon were used to estimate approximate thermoneutral zone, as well as lower critical temperature and upper critical temperatures. The final core body temperature ranges used were derived from those determined by the clumped isotope paleothermometry analysis, as well as published core body temperatures from extant xenarthrans (McNab 1985; Cliffe et al. 2018; Seitz and Puig 2018). Output thermoneutral zones were determined within a target range of 5% ± basal metabolic rate with the animal modeled as standing still with no exposure to sunlight. Constant wind speeds of 0.1 ms^− 1^ were also assumed for the metabolic chamber simulations. The upper critical temperature was determined when the metabolic rate fell below 5% of the target basal metabolic rate range, while the lower critical temperature was determined when the metabolic rate was above 5% of the target basal metabolic rate range as per previous analyses (Lovelace et al. 2020). This process was repeated for each proposed integument model.

To account for environmental conditions of the fossil localities (beyond ambient temperatures) during the Pleistocene, the Paleobiology Database (https://paleobiodb.org) was used to determine the northernmost and southernmost ranges, in addition to a mid-latitude species range to test for a variety of climatic conditions for each taxon. Average yearly temperatures corresponding to the approximate stratigraphical ages of the simulated localities were obtained from previously published PALEO-PGEM v1.0 simulations of various global mean annual temperatures over a span of 5 million years with a total of 5,001 measurements in 1,000-year increments (Holden et al. 2019). The global climate extractor in Niche Mapper was then used to determine the factored difference from modern climate values of monthly maximum and minimum temperature for the estimated stratigraphical age of the locality. Relative humidity, cloud cover, and wind speed were approximated from modern values measured by global climate extractor database in Niche Mapper for similar regions. The microclimate model in Niche Mapper converts these user-specified monthly max/min climate data into hourly values using sinusoidal functions that assume the same weather conditions for each day of a given month. The model then solves for heat and water mass balance for the ground surface and the temperatures, wind speeds, and humidity (2 m above and below ground level). Solar radiation is computed from an embedded solar radiation algorithm (McCullough and Porter 1971). Sky thermal radiation was computed from an empirical regression based on the input for the 2 m above ground air temperature (Idso and Jackson 1969).

Finally, for the Niche Mapper simulations of ground sloths in their respective environments, a thermal comfort zone was determined for the estimated daily metabolic rate requirements for each taxon based on likely upper and lower bounds of daily metabolic activity. Total daily metabolic expenditure was calculated using a trapezoidal integration of hourly metabolic rate predictions. The lower bound of the target zone was determined by multiplying estimated basal metabolic rate in kJ/hr^− 1^ by 24, while the upper bound was determined by multiplying estimated field metabolic rate (i.e., basal metabolic rate multiplied by a factor of 2 converted to kJ/hr^− 1^) by the same factor. Within this zone, the simulated animals could function at normal metabolic levels as long as resources were readily available. For simplicity, zones of tolerance and survival above the upper bounds of the upper limits of the modeled thermal comfort zone are referred to herein as “cold stress” since the simulated animal would be expending additional metabolic energy to prevent hypothermia. Likewise, zones of tolerance and survival below the lower bounds of the thermal comfort zone are referred to as “heat stress” as the simulated animal would have to lower its metabolic rate below its estimated basal metabolic rate to prevent hyperthermia. The results of Fariña (2002) provided a sensitivity analysis to compare the thermal tolerances of ground sloths with metabolic rates more akin to large placental mammals, as opposed to a xenarthran-like metabolism.

### Statistical analysis

Predictions of daily metabolic rate (in kJ day^− 1^) that were generated by the climate simulations of each integument (fur coverage) model were plotted as a series of curves to determine which model best fit the estimated minimum and maximum daily metabolic rate expenditures, or the hours per day where basal metabolic rate can be maintained without the need for seeking shade, for each taxon. Once the integument models with the least amount of thermal stress were established, estimates of maximum hours of metabolic activity beyond levels of basal metabolic rate were compared across the geographic distribution of each taxon along with those of extant placental mammals of similar size ranges. Daily food consumption averages in kg/day^− 1^ extrapolated from integument models with the least amount of thermal stress for each simulated environment were fitted with a second-order polynomial regression curve to indicate possible differences in food intake among taxa. Nutritional value of leaves, grasses, and woody plants were derived from the DairyOne feed composition library (https://apps.dairyone.com/feedcomposition/; see Online Resource 1 for full list of climate and physiology parameters), and the wet mass in kg of forage consumed was additionally estimated from these simulations. The consumed food data were also fitted with second order polynomial regressions to statistically evaluate patterns of monthly food consumption in the respective predicted habitats of the sampled fossil taxa.

## Results

### Clumped isotope paleothermometry and REE ratios

Calculated REE index ratios for a majority of specimens analyzed here fell below a value of 1.0 indicating little diagenetic alteration in the samples (Table 1). An exception was the *Promegatherium* specimen (FMNH P14511), which yielded a REE index value of 36.6 and was considered to be likely diagenetically altered or an impure sample. The *Eremotherium* specimen (UF 312730) had the lowest REE ratio (0.02) that is consistent with both dentine to cortical bone REE index values determined for other individuals found at Halie 7G (range; 0.004–0.01; MacFadden et al. 2010; Online Resource 2). Both the *Eremotherium* (REE ratio; 0.42) and *Nothrotheriops* (REE ratio; 0.86) tooth specimens recovered from Inglis 1 A and Leisey 1 A, respectively, also fell within the known variation of REE index values recorded for fossils from individuals at both localities (range; 0.33–0.72; MacFadden et al. 2010). The corresponding clumped isotope data from the *Promegatherium* specimen FMNH P14511 resulted in a mean temperature estimate of 23 ± 5° C and was determined to be too low for a realistic body temperature of a large mammal, being more similar to the lower end of body temperature estimates of ectotherms determined by similar previous analyses (Eagle et al. 2011, 2015; Griffiths et al. 2023). It is noted that the carbonate-phosphate δ^18^O offset was within the bounds of most extant vertebrates, indicating that alteration of the carbonate moiety may be subtle rather than extensive (Fig. 1b).

Despite its low REE index, the *Eremotherium* specimen UF 312730 exhibited poor replication between analyses (high standard error; Table 1) and yielded clumped isotopic temperature estimates which are implausible to be in a physiologic range. This was likely the consequence of organic contamination that had not successfully been removed by pre-cleaning of the tooth specimen and/or incomplete CO_2_ cleanup stages of sample purification, as neither phosphate δ^18^O and REE indicated diagenesis (Table 1; Fig. 1b). This hypothesis is supported by the observed abnormally high Δ_48_ values (Fig. 1c; Online Resource 2) in this sample indicative of the presence contamination creating isobaric interferences for clumped isotope measurements. The three remaining teeth specimens of *Eremotherium*, *Nothrotheriops*, and *Megatherium* that had low REE and carbonate-phosphate δ^18^O offsets in the range of extant mammals (Table 1; Fig. 1b) yielded clumped isotope temperatures of 29 ± 2° C, 32 ± 3° C, and 31 ± 1° C, respectively, with good agreement between replicate analyses and relatively low Δ_48_ values (Fig. 1c). As per established criteria, there is no reason to suspect that these samples are compromised by diagenesis or analytical artifacts, thus the resulting estimates are likely to represent the core body temperatures of extinct ground sloths.

### Estimates of paleoclimate and physiological parameters

**Fig. 3:**
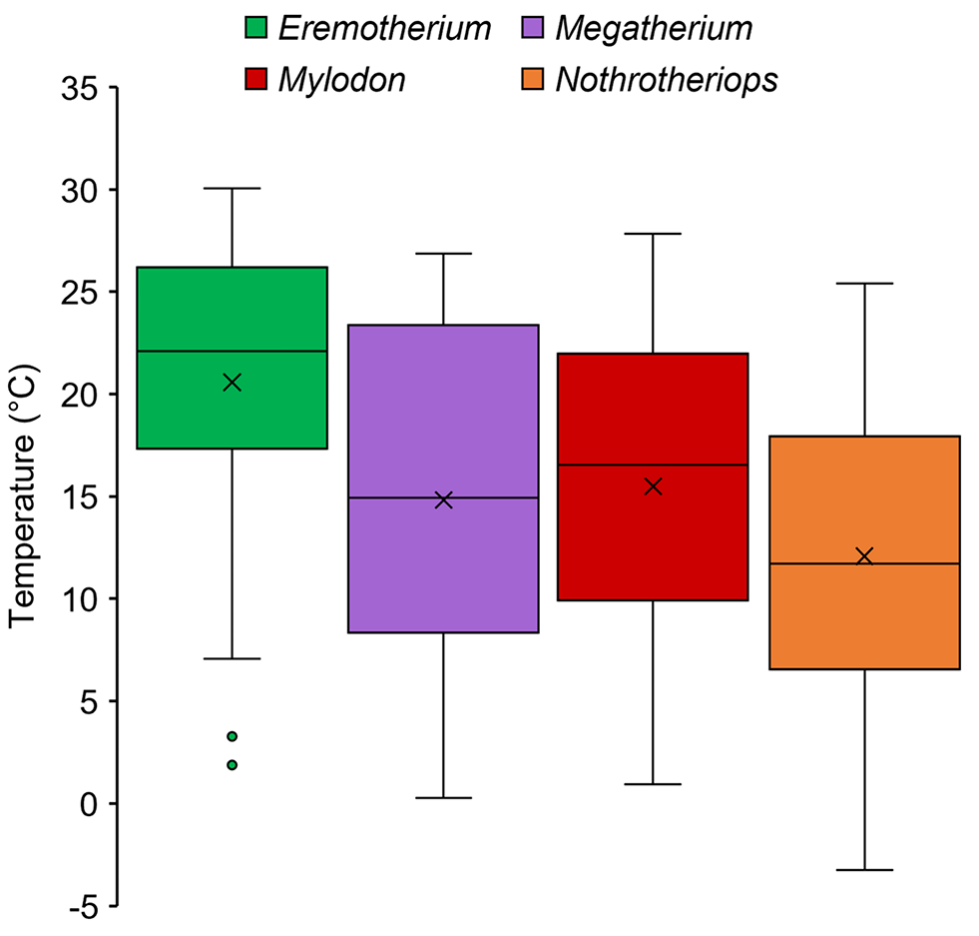
Box and whisker plot of ambient temperature estimates for the environments inhabited by the four extinct ground sloth taxa sampled

**Fig. 4:**
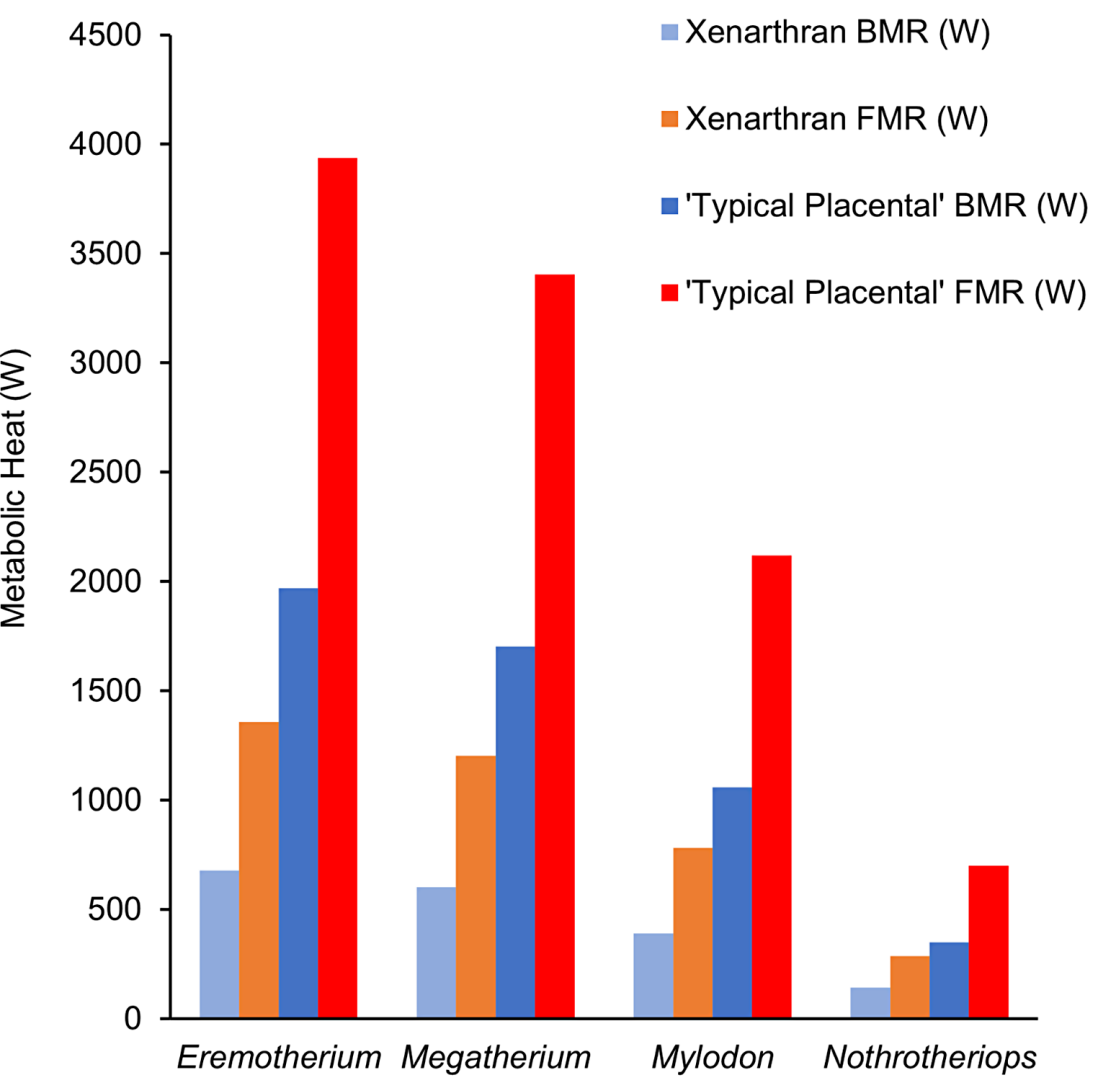
Bar chart of estimated basal metabolic rate (BMR) and field metabolic rate (FMR) in ground sloths. Values in unit watts (W) calculated from the xenarthran metabolic scaling equation (White and Seymour 2004) are compared to those (right) based on a ‘typical placental’ metabolism (Kleiber 1932; Fariña 2002)

A wide range of ambient temperatures were modeled for the four genera of ground sloths analyzed (Fig. 3). The largest, *Eremotherium*, was predicted to have experienced variable ambient temperatures (range: 1.89–30.1º C) throughout its extensive geographic range, though the lowest values were determined by outliers. The predicted ambient temperature differences for *Nothrotheriops* were similar in absolute values to *Eremotherium*, but colder minimum ambient temperatures were estimated for this taxon (range: -3.2–25.4º C). Both *Megatherium* and *Mylodon*, however, were predicted to primarily have inhabited cold-to-temperate environments with minimum and maximum temperature values overlapping with the temperature ranges for *Eremotherium* and *Nothrotheriops* (Fig. 4). Estimated basal metabolic rate values for all four taxa sampled did not exceed 1,000 W (Fig. 4); all metabolic rate estimates were 59–66% less than those approximated by previous analysis of Fariña (2002).

### Metabolic chamber simulations and thermoneutral zone ranges

**Fig. 5:**
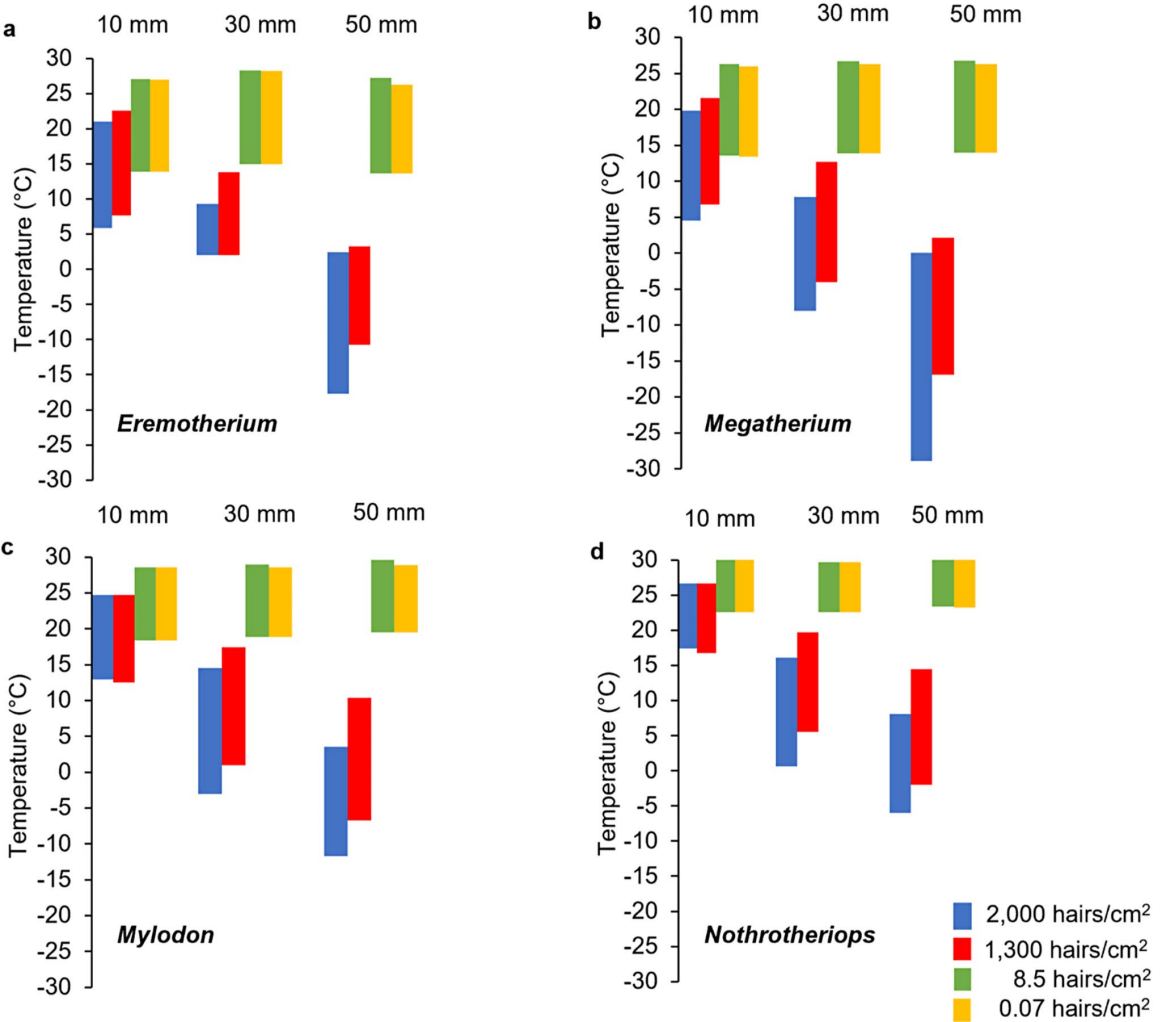
Thermoneutral zones of **a**. *Eremotherium*; **b**. *Megatherium*; **c**. *Mylodon*; and **d**. *Nothrotheriops*. Estimated thermoneutral zones were derived from metabolic chamber simulations with different fur densities and coat thicknesses

Metabolic chamber analyses revealed broad thermoneutral zones for dense fur (1,300–2,000 hairs/cm^2^) across all four genera that decreased with an increase in coat thickness (Fig. 5). Both *Eremotherium* and *Megatherium* had similar thermoneutral zones with coat thicknesses of 10 mm (range; 4.5–22.6° C, combined for both taxa), which overlap with the estimated ambient temperature values that both genera would have experienced throughout their geographic distribution (Fig. 3). Surprisingly, *Megatherium* with a dense fur and a coat thickness of 50 mm had the lowest lower critical temperature among the four genera (-27° C) that is equivalent to previous estimates for a densely furred *Mylodon* within its thermoneutral zone (Fariña, 2002), but lower than lower critical temperature estimates of *Eremotherium* with the same integument features applied herein (-18° C). Dense fur conditions for *Mylodon* and *Nothrotheriops* with coat thicknesses of 10–50 mm also have thermoneutral zones that overlap with likely ambient temperature ranges that each taxon would have experienced, as well as ambient temperatures well beyond the lowest estimates modeled here (-12–24° C and − 6–26.7° C, respectively). Consistent trends of narrow thermoneutral zones resulting from sparse fur (0.07–8.5 hairs/cm^2^) with lower critical temperature estimates higher than 10° C and upper critical temperature estimates ranging from 27–30° C were determined for all four taxa analyzed.

### Microclimate simulations and integument thermal tolerances

**Fig. 6:**
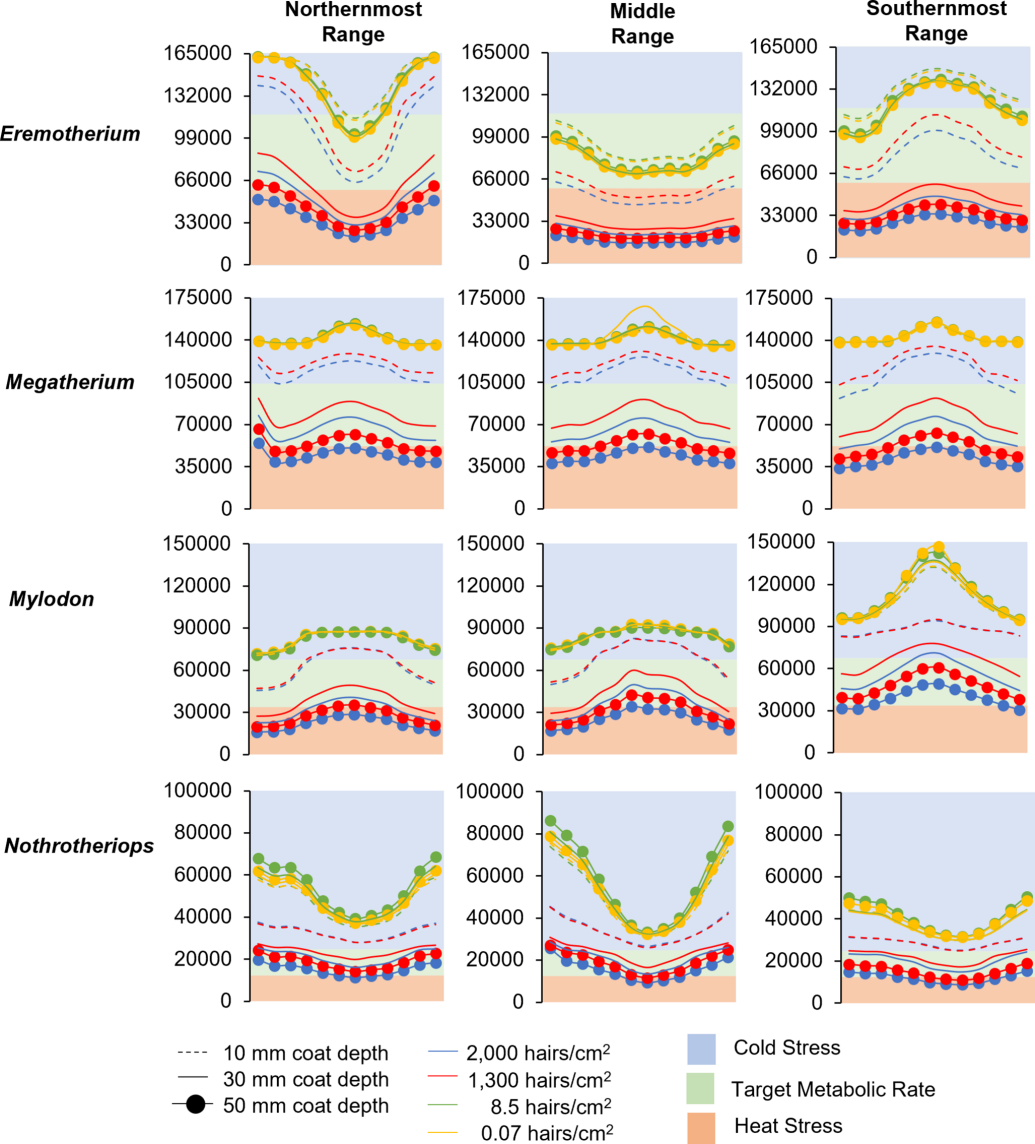
Modeled trends of ground sloth daily metabolic activity (kJ day^− 1^; *y*-axis) in response to climatic factors over the course of a year (month; *x*-axis) across the northernmost, middle, and southernmost range of each genus. *Eremotherium* target metabolic rate = 58,646–117,293 kJ day^− 1^; *Megatherium* target metabolic rate = 51,944–103,887 kJ day^− 1^; *Mylodon* target metabolic rate = 33,765–67,530 kJ day^− 1^; *Nothrotheriops* target metabolic rate = 12,372–24,745 kJ day^− 1^

Similar outcomes favoring dense fur coverages were generally observed when multiple climatic variables, including relative humidity, precipitation, wind speed, and ambient temperature were input in the refined paleoclimate models. Notably, *Eremotherium* was estimated to have no thermal stress in all environments with dense, 10 mm fur insulation (1,300–2,000 hairs/cm^2^), but would have experienced prominent cold stress in the northernmost range of its distribution, although no thermal stress occurring in the southernmost latitudes during the coldest months of the year in both localities sampled (Fig. 6). This result contrasts with the resolved trends that sparse fur coverage indicated in northernmost and southernmost latitudes, which consistently showed no thermal stress during only the warmest months of the year. Dense 30–50 mm fur insulation for *Eremotherium* was determined to be mostly detrimental by resulting in overall heat stress, except for during the winter months in only the northernmost latitudes and year-round in the southernmost latitudes. Conversely, *Eremotherium* additionally was modeled to have no thermal stress with sparse 50 mm fur, but with more dense fur coverage resulting in constant heat stress in neotropical regions (Fig. 6).

The refined paleoclimate models showed that *Megatherium* would have avoided thermal stress year-round with full body, dense 30 mm fur insulation in all three of the simulated environments, whereas full body, sparse fur (both depths and densities) resulted in cold stress throughout the year (Fig. 6). Both *Mylodon* and *Nothrotheriops* would also have avoided any thermal stress with dense fur insulation (density; 1,300–2,000 hairs/cm^2^). In particular, *Mylodon* was modeled to have no thermal stress with 10 mm fur during the warmest months of the year and also with 30 mm fur during the coldest months at northern and mid latitudes (Fig. 6). Both *Mylodon* at the southernmost extent of its range and *Nothrotheriops* across its entire geographic range were determined to have no heat stress with dense fur with a coat thickness of 50 mm.

### Daily hours of active metabolic expenditure

**Fig. 7:**
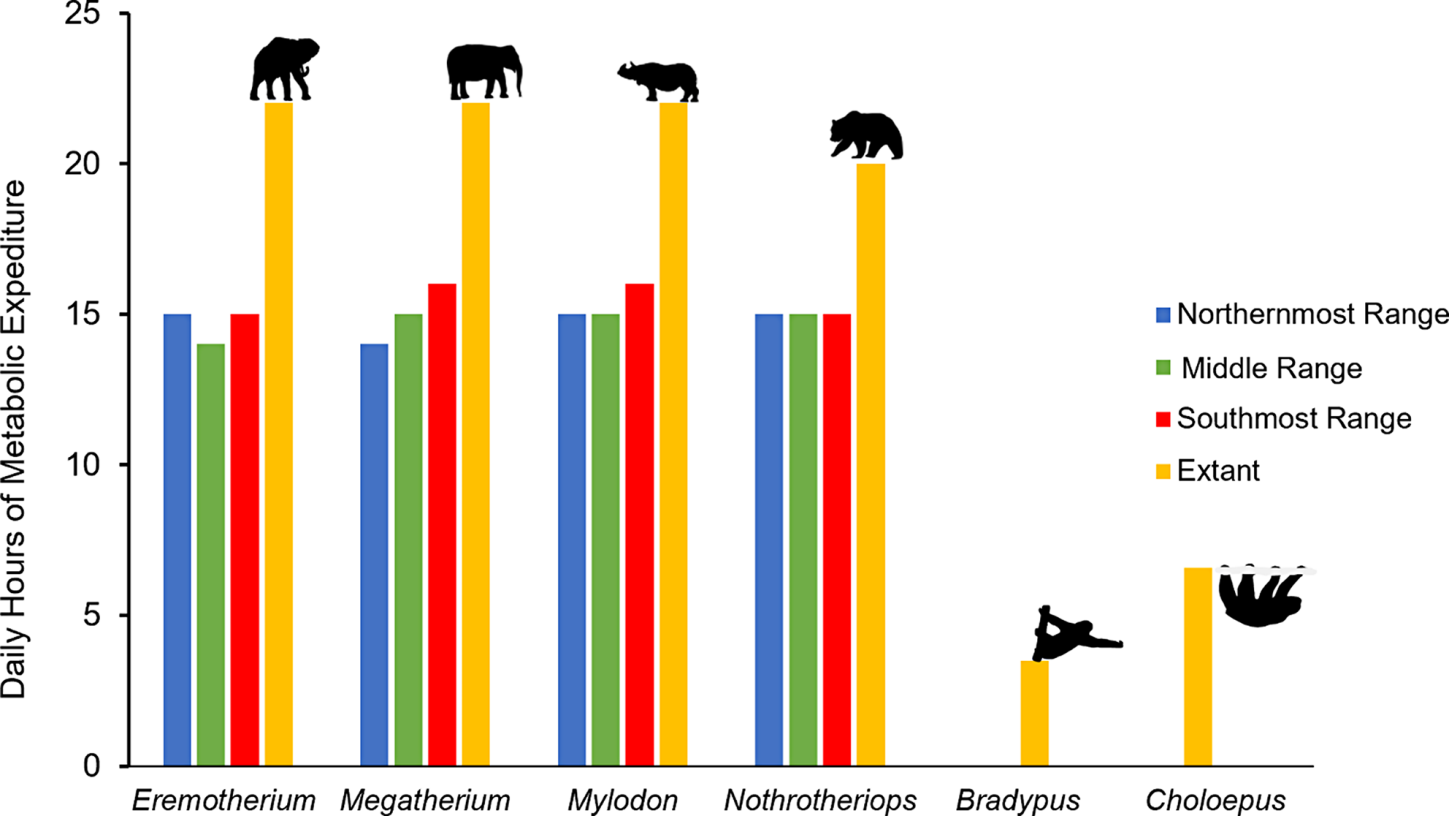
Bar chart of the average number of hours per day where metabolic rate can surpass basal levels in the northernmost middle, and southernmost range of each ground sloth genus compared to extant placental mammals of similar body sizes and extant tree sloths. Shown as silhouettes are the body size analogues in order left-to-right: African savanna elephant (*Loxodonta africana*; for *Eremotherium*), Asian elephant (*Elephas maximus*; for *Megatherium*), Indian rhinoceros (*Rhinoceros unicornis*; for *Mylodon*), brown bear (*Ursus arctos*; for *Nothrotheriops*), *Bradypus* and *Choloepus*. Silhouettes were obtained from PhyloPic.org (Michaud M, Taylor J, Keesey M, Morrow C, Jenkins X) and are under public domain (Total hours of activity for extant analogues were based on daily hours of sleep reported in the literature (Stelmock and Dean 1986; Deka and Sarma 2015; Gravett et al. 2017; Cliffe et al. 2023)

Estimates of hours per day where basal metabolic rate can be maintained without seeking shade for the integument conditions resulting in the least thermal stress (by fur coverage, depth, and density) across the known geographic distributions of each taxon analyzed averaged approximately 15 h day^− 1^ (Fig. 7). While the megatheres were modeled to have large metabolic expenditure in cold climates (e.g., northern geographic ranges of *Eremotherium*; mid-latitudinal and southern geographic ranges of *Megatherium*), the greatest number of hours of metabolic activity expended per day (= 16 h day^−^1) were predicted in the southern latitudinal ranges of both *Megatherium* and *Mylodon*. *Nothrotheriops* showed a consistent maximum metabolic activity value of 15 h day^− 1^ throughout its geographic range (Fig. 7). Comparative data on daily energetic expenditures indicate that extant body size analogues of *Eremotherium*, *Megatherium*, and *Mylodon* (e.g., African elephant, Indian elephant, and Indian rhinoceros, respectively) all have values of 22 h day^− 1^ (Deka and Sarma 2015; Gravett et al. 2017) while that for much smaller *Nothrotheriops*, a brown bear (*Ursus arctos*) analog, was measured at 20 h day^− 1^ (Stelmock and Dean, 1986). In sum, all ground sloth taxa were predicted to have 36.4–31.9% less metabolic activity than modern placental mammalian analogues of similar body masses, but are far more active than extant tree sloths (e.g., 3.48–6.48 h day^− 1^; Cliffe et al. 2023).

### Monthly dietary intake

**Fig. 8:**
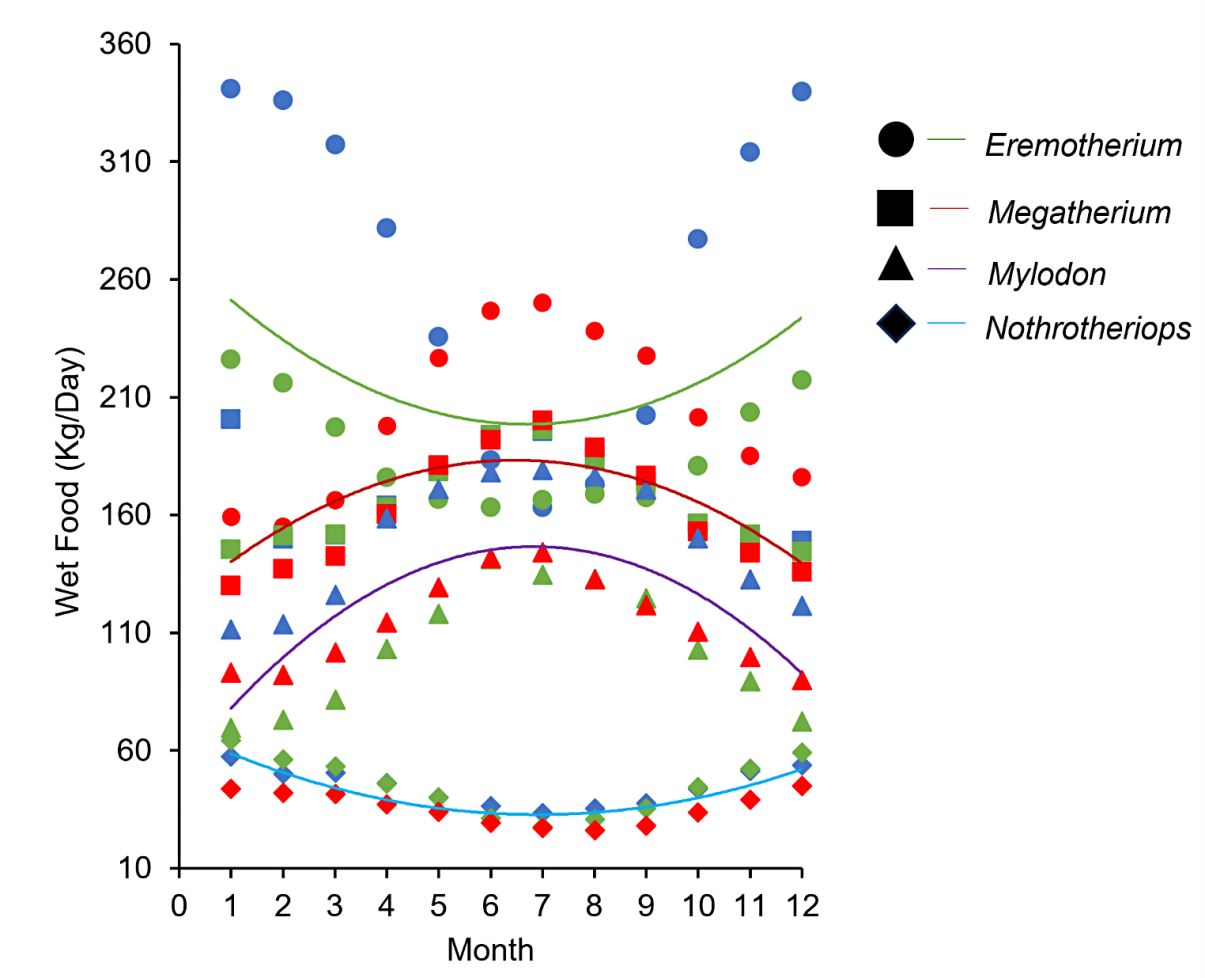
Scatter plot of kilograms of wet food mass consumed per day throughout the year for each integument model exhibiting minimal thermal stress for northernmost (blue), middle (green), and southernmost ranges (red). Data for each genus of ground sloth are fit with second order polynomial regressions: *Eremotherium* (y = 1.6186 × 2–21.704x + 271.31, R² = 0.097); *Megatherium* (y = -1.4345 × 2 + 18.586x + 123.05, R² = 0.535); *Mylodon* (y = -2.017 × 2 + 27.553x + 52.53, R² = 0.513); *Nothrotheriops* (y = 0.7428 × 2–10.255x + 68.247, R² = 0.669)

The estimated distributions of energy intake across all four genera indicated no statistically significant trends in monthly food consumption with a change in environment (Fig. 8). While a general increase in food consumption was demonstrated for the large-bodied megatheres, *Eremotherium* was modeled to experience little-to-no increase in food consumption (R^2^ = 0.009) throughout its wide geographic distribution. The genus *Megatherium* was modeled to experience a moderate increase in monthly food intake in the southernmost latitudes (R^2^ = 0.53), whereas the simulations of *Mylodon* predicted a similar increase in food intake in southern latitudes (R^2^ = 0.51), notably overlapping with that of *Megatherium*. Finally, the data trends for *Nothrotheirops* showed a moderately strong decrease in monthly food intake in the southern latitudes (R^2^ = 0.67) during the summer months.

## Discussion

### Geochemistry of fossil teeth and interpretations of physiology

The similarities in clumped isotope temperatures from teeth recovered from different locations, coupled with the low REE index values, provides confidence that these estimates may reflect the actual core body temperatures of extinct ground sloths. These temperatures are notably lower than the clumped isotope derived temperatures of other large mammals including values of approximately 37–39° C for an Indian elephant and extinct mammoths (Eagle et al. 2010), and ~ 34° C for a baleen whale (Griffiths et al. 2023). Tooth material from ground sloth specimens showing little diagenetic alteration were obtained from three different depositional environments and produced temperature estimates within the range of extant xenarthran body temperatures. Given that the estimates for *Nothrotheriops* were derived from a single tooth sample with a relatively high REE ratio (0.86), some degree of caution should be given to the interpretation of the results from this specimen. Measurements obtained from the *Eremotherium* sample UF 312730 yield higher values in both Δ47 and Δ48 which could indicate contamination of the samples resulting in isobaric interference and a subsequent overestimation of Δ47 and unrealistically cold Δ47 derived temperatures (Fig. 1c). This aside, these novel data suggest that extinct ground sloths may have been heterothermic, and likely fluctuated their core body temperatures as a main means of thermoregulation. The core body temperature estimates derived from the clumped isotope analyses are indeed within the range of error of known body temperatures for extant xenarthrans (30.2–36.6° C), further verifying body temperature values determined for the Niche Mapper simulations and suggesting that the observed ubiquitous similarities in thermophysiology are an ancestral trait in this clade.

Clumped isotope estimates of body temperature, however, are on the lower end of Niche Mapper simulations for the mean estimate of 29 ± 2° C determined for UF 95869. This could be explained by the measurements taken from specific tooth loci reflecting potential periods of torpor akin to what has been proposed for troodontids (Tagliaventoor et al. 2023), estimates from teeth might may also reflect slight differences in core body temperature and the temperature of tooth mineralization as has been explored in interpreting clumped isotope values derived from regionally endothermic sharks (Griffiths et al. 2023). These hypotheses should be explored in future analyses focusing on taking samples from specific regions of internal orthodentine to determine if core body temperatures were fluctuating throughout the life of the animals. Such methods were previously applied to teeth from *Eremotherium* to infer the environmental conditions during the life of a single individual, specifically using δ^13^C and δ^18^O measurements (Larmon et al. 2019). Analyses of more specimens spanning a broader range of climate conditions similar to the approaches that have been used on non-avian dinosaur remains (Fricke and Rodgers 2000; Amoit et al. 2006) may also be informative. Nevertheless, the clumped isotope measurements along with the results of the physiological modeling analyses herein collectively indicate that extinct ground sloths had lower body temperatures and basal metabolic rates than most large terrestrial placental mammals.

### Comparisons with previous analyses

The low body temperature estimates obtained from fossil ground sloth teeth suggest lower metabolic rates akin to extant xenarthrans and contradict the higher values of metabolic rates based on estimated blood flow rate (Varela et al. 2024). Varela et al. (2024) hypothesized that small nutrient foramen diameters of the femur, and thus restricted blood flow rates, along with the fossorial and arboreal habits of extant xenarthrans all correlated with low metabolic rates. However, raw data from that study shows overlap between femoral nutrient foramen diameters and blood flow rates of extant xenarthrans and placental mammals with elevated metabolic rates. For example, the nutrient foramina of both the giant anteater (*Myrmecophaga tridactyla*) and the leopard seal (*Hydrurga leptonyx*) have a measured diameter of 0.9 mm despite their extremely different functional habits and ecology. Given these types of discrepancies in their analysis, we caution that using measured nutrient foramen diameter alone is not a reliable predictor of metabolic rate, and it could likely reflect an artifact of similar body size. The core body temperature estimates obtained from the established clumped isotope analyses used herein potentially offer a more direct estimation of core body temperature and expand our understanding of the physiological limitations potentially experienced by extinct ground sloths.

The paleoclimate simulations also yielded metabolic rate values drastically different values from those of the Fariña (2002) study on ground sloth thermal energetics. In addition to refined estimates of clade specific basal metabolic rates for ground sloths, numerous other environmental factors beyond ambient temperatures were input and modeled across the geographic distributions of each genus to determine more exact boundaries of thermal neutrality. The basal metabolic rate estimates derived from the xenarthran scaling equation (White and Seymour 2004) produced metabolic rates representing only 34–41% of those for mammals with typical placental metabolism as determined by the relationship of Kleiber (1932) (Fariña 2002). Substantially lower basal metabolic rates were predicted in the present study, thus producing markedly different results for thermal tolerance ranges across the various integument conditions tested compared with those already reported. The main previous study (Fariña 2002) proposed that a sparsely furred *Megatherium* with a body mass of 4,000 kg and metabolism that was 50% that of a typical placental mammal would be within its thermoneutral zone at ambient temperatures of 10° C versus the results from the present climate simulations, which suggest that *Megatherium* under similar conditions would have been cold stressed.

Traditional determinations of thermoneutral zones using the xenarthran metabolism as a proxy suggest *Eremotherium* and *Megatherium* with dense fur and a coat depth of 10 mm would be akin to those of sparsely furred models (see Fig. 5). However, the microclimate models employed herein demonstrated that environmental factors aside from ambient temperature (i.e., relative humidity, wind speed, etc.) and metabolic activity (i.e., locomotion, foraging, etc.) accounted for the predicted outcome that dense 10 mm thicknesses would benefit *Eremotherium* only during the warmest months of the year at its northernmost and southernmost ranges year-round. The novel microclimate models also showed that having dense fur at a coat thickness of 10 mm would result in constant heat stress for *Eremotherium* in the neotropics, yet constant cold stress for *Megatherium* throughout its geographic distribution (Fig. 6). Despite heat stress modeled to be present in the tropical populations of *Eremotherium* having a full body fur coverage at a depth of 30–50 mm, the condition of dense 10 mm fur depths would allow it to exhibit no intense thermal stress year-round, even during the hottest months of the year throughout its entire geographic range. The findings for southernmost region for *Mylodon* and those data for *Nothrotheriops* throughout its entire range with dense 50 mm fur depths, suggest nearly complete yearly protection from thermal stress in their respective environments, with mild cold stress for the three coldest months of the year (predicted temperatures; -3.26–0.9° C). These results also contrast with previous estimates (Fariña 2002) that thermal neutrality for *Mylodon* having a basal metabolic rate half of the typical placental metabolism and dense fur 40 mm deep would be at an ambient temperature of -28° C.

### Megathere thermal neutrality and geographic distribution

**Fig. 9:**
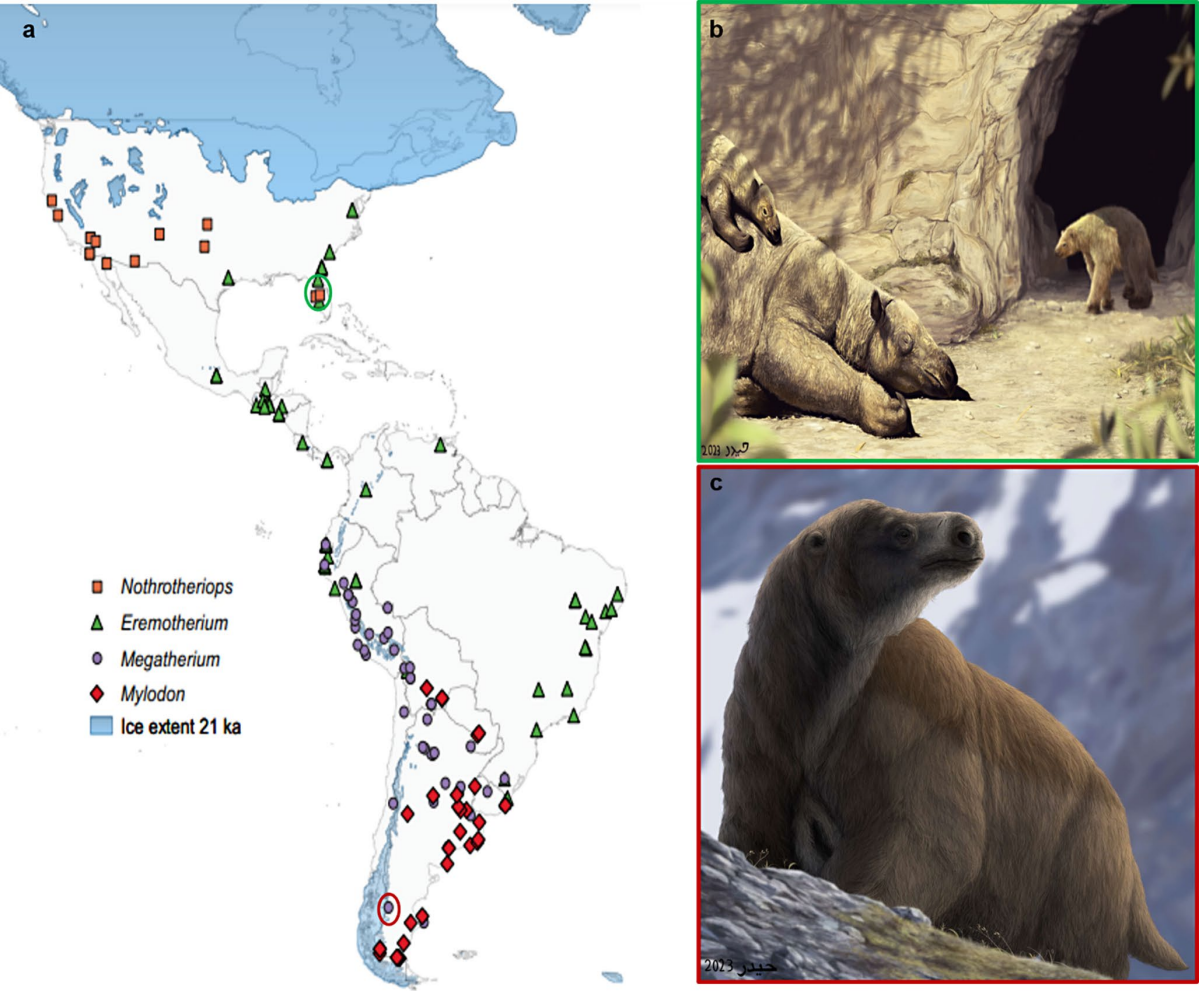
**a**. Map of geographic distribution for Pleistocene-aged sites of fossil remains of ground sloths *Eremotherium*, *Megatherium*, *Mylodon*, and *Nothrotheriops* spanning from 2.5 MYA to 11,000 years ago. Areas shaded blue represent the extent of glaciers during the last glacial maximum 21,000 years ago and red and green circles indicate locations where the paleo art is depicted. Data: political boundary base map was generated using QGIS3’s ‘World Map’; hand-drawn ice sheet overlay informed and modified after published data (Ehlers and Hughes 2018); spatial data for species distributions was retrieved from the Paleobiology Database (paleobiodb.org). **b**. Reconstruction of an adult and infant *Eremotherium* going through seasonal fur shedding while wallowing in a mudhole outside the cave den of an emerging *Nothrotheriops* in the early spring of Florida during the Pleistocene (location indicated by green circle in panel 9a). **c**. Reconstruction of *Megatherium* enduring the cool, windy Pampas of Argentina with a thick fur coat (location indicated by red circle in panel 9a). Paleoart by Syed Jaffri H

The data from the paleoclimate analyses revealed critical insight into the thermoregulatory thresholds of extinct ground sloths and how these would have impacted their life appearance by condition of their integument. In particular, the results for *Eremotherium* suggest that a wide range of integument models could be plausible depending on ambient temperatures of their past environments. Given that *Eremotherium* had a geographic distribution spanning as far north as Virginia in North America and as far south as Brazil in South America, it is plausible that this genus had varying degrees of fur coverage throughout its geographic range. Sparse, full body fur coverage would have been beneficial at mid-ranges with no thermal stress experienced year-round. Changes in fur depth would also have been beneficial during warmer months for *Eremotherium* at the most northern extent of its range, although there is no evidence of coat shedding in extant xenarthrans. Nonetheless, dense 10 mm fur could have prevented thermal stress at average ambient temperature ranges of 5.9–22.6° C that *Eremotherium* was expected to have experienced (Fig. 6). Moreover, because *Eremotherium* crossed the Isthmus of Panama into North America but subsequently retreated to South America during the last glacial maximum (McDonald and Lundelius 2009) it is possible for it to have undergone migrations during times of extreme seasonality. Due to the modeled presence of mild cold stress with dense 10 mm fur and no thermal neutrality with a 50 mm fur depth in the southernmost ranges, it is therefore suggested that *Eremotherium* was well-adapted to the neotropical conditions expected to be prevalent in the southernmost extent of its range (Fig. 9a). This hypothesis notably agrees with the absence of *Eremotherium* in North America during the last glacial maximum.

Despite being similar in body size to *Eremotherium*, the genus *Megatherium* is expected to have exhibited constant cold stress with only sparse fur coverage throughout its geographic range. This can be explained by *Megatherium* being known to have inhabited cooler, dry environments in the pampas of Argentina and Chile. Using similar modeling approaches to physiology as presented herein, previous sensitivity analyses (Lovelace et al. 2020) revealed that ambient temperature and wind speed values were the two climatic factors that most strongly influence predicted values of field metabolic rate, whereas the variables of relative humidity and cloud coverage had little effect on simulated metabolism. Thus, dense fur for *Megatherium* might have been needed to counteract greater exposure to the elements (e.g., wind, rain, or snow) in more open environments (Fig. 9c). This integument condition is reflected in the present climate simulation results which indicate that with a full body coverage of fur at depths of 10 mm, *Megatherium* would have been constantly cold-stressed regardless of fur density, but it would have complete thermal neutrality in all latitudinal distributions with dense (1,300–2,000 hairs/cm^2^) fur with a coat depth of 30 mm. Conversely, by having dense 50 mm fur, *Megatherium* would have been constantly heat-stressed by over insulation (Fig. 6). Furthermore, considering that fur depths of 30–50 mm resulted in predicted heat stress for similarly-sized *Eremotherium* (with the exception of the coldest months at northern extents of its range), it is reasonable to speculate that neotropical conditions may have acted as a thermal gradient restricting *Megatherium* to southern latitudes and occupation of the westernmost regions of Peru and Ecuador (Fig. 9a; Pujos and Salas 2004). This hypothesis is supported by models of environmental distributions of various Pleistocene xenarthrans by Varela et al. (2018) which demonstrate little-to-no overlap between the geographic ranges of *Eremotherium* and *Megatherium* suggesting distinct differences in habitat preference.

### Thermoregulation in *Mylodon* and *Nothrotheriops*

Both *Mylodon* and *Nothrotheriops* were modeled to require dense 10–50 mm fur depths across their respective ranges of predicted ambient temperature exposure, which is directly observed in the preserved pelts from both genera (Ridgewood 1901; Hausman 1929). Nevertheless, given that *Mylodon* was known to inhabit the Andes Mountains in South America, and *Nothrotheriops* would have experienced paleoclimate temperatures as low as -3.3° C (Fig. 3), behavioral thermoregulation would have been expected in cold climates in addition to having thick fur coverage. One supposition is that thermal neutrality could have been maintained by taking shelter in caves during periods of thermal stress. Mean annual temperature estimates of caves in the American southwest where remains of *Nothrotheriops* have been documented reveal stable temperature ranges that would have provided protection against periods of thermal stress (6.6–17° C; McDonald 2022; Fig. 9b). Localities for *Mylodon* in Chile such as Chica cave exhibit a similar range of stable temperatures (4.83–17.4° C; Nehme et al. 2022). A second hypothesis is that both taxa may have undergone periods of torpor and/or hibernation as a behavioral means of cold tolerance.

Isotopic and taphonomic studies on the extinct Patagonian ‘panther’ (*Panthera onca mesembrina*) revealed that bite marks attributed to this extinct predator have been found on the bones of juvenile and adult individuals of *Mylodon* in Cueva del Milodón (Prevosti and Martin 2013; Martin 2018). While *Mylodon* was smaller than the megatheres, an adult individual was still much larger than a jaguar, which suggests that individuals found with bite marks in caves were preyed upon while they were undergoing torpor, when they would have been most vulnerable to attack. Additional support for the above hypotheses proposed herein can also be linked to the behavioral thermoregulation strategies observed in modern armadillos. For example, the nine-banded armadillo (*Dasypus novemcinctus*) is known to seek shelter during periods of extreme cold and employ a combination of torpor with episodic foraging (McBee and Baker 1982; Knight et al. 2020). Regular torpor also has been documented in the pichi armadillo (*Zaedyus pichiy*) (Superina and Boily 2007; Superina and Abba 2014), and it is the only known extant xenarthran to undergo true hibernation.

It is also possible that *Mylodon* and *Nothrotheriops* may have had higher activity levels than those estimated in the present analysis. However, higher metabolic demands in the smaller ground sloths would only be useful in temperatures well below the range of ambient temperatures predicted for both taxa. Accordingly, another potential explanation for their thermal energetics involves having a diet richer in energy than their megathere conspecifics. A recent analysis of amino acids (Tejada et al. 2021) suggested that the diet of *Mylodon* could have consisted of animal protein due to the nitrogen signatures found in their fossilized bone material. Though the exact timings of nitrogen signatures indicative of omnivory could not be determined, animal protein consumption would have had thermoregulatory benefits for *Mylodon*. A protein-rich diet provides some flexibility to thermal neutrality in cold climates, and it is possible that *Mylodon* consumed animal matter sporadically during the coldest months, in addition to foraging on grasses (Varela et al. 2023). Conversely, nitrogen isotopes recovered from *Nothrotheriops* fossil remains definitively did not show evidence for an omnivorous diet, but instead, this genus was eating carbohydrate-rich desert plants such as globemallow and casava root (Poinar et al. 1998).

Mylodontids also possessed dermal ossicles of varying shapes and sizes which may have provided protection against predators (Collins 1933; McDonald 2018; Toledo et al. 2021). Given the vascularized nature of these structures in ground sloths as a whole, Cartelle and Bohórquez (1986) hypothesized that the dermal ossicles may have played a role in thermoregulation as well. Yet, histological examinations of *Mylodon* ossicles by Toledo et al. (2021) show no features indicating the storage of adipose tissue akin to those in extant armadillos (Krmpotic et al. 2009). While measurements of the thermal conductance have yet to be performed, Toledo et al. (2021) implied that the fur of mylodontids such as *Mylodon* would have provided a greater amount of insulation compared to vascularized osteoderms.

### Integument and behavioral factors

Giant ground sloths have historically been reconstructed as being densely furred taxa and the novel data provided by the Niche Mapper simulations do little to challenge this integument appearance. In contrast, the modeling results disagree with more sparsely furred, elephant-like integument reconstructions of *Eremotherium* and *Megatherium* (Fariña 2002). Despite the obvious discrepancies between studies on thermal neutrality and its effects on ground sloth integument, several critical behavioral and ecological preferences can now be inferred. For example, dental microstructure and microware patterns (Green and Kalthoff 2015), in addition to isotopic analyses (Dantas et al. 2021), suggest a completely herbivorous diet for *Eremotherium* and *Megatherium* despite previous speculation that they were possibly omnivorous and/or engaged in scavenging behavior (Fariña and Blanco 1996). Indeed, the diets of both taxa would have primarily consisted of leaves and other high-growing vegetation (Bargo et al. 2006; Green and Kalthoff 2015; Bocherens et al. 2017), which overlap with those of their closest extant relatives *Bradypus* spp. (Cliffe et al. 2015; Pauli et al. 2016; Preslee et al. 2019; Delsuc et al. 2019; Tejada et al. 2024). Leaves are poor in nutritional value correlating with the extremely low metabolic rates observed in *Bradypus variegatus* (Cliffe et al. 2015; Pauli et al. 2016) and may be a primary factor for why megatheres would have had low metabolic rates for mammals of their massive body size.

One of the main outcomes of our is that megatheres are predicted to have a basal metabolic rate more similar to modern xenarthrans than other placental mammals. Yet, they also could have exhibited a number of behaviors similar to large extant mammals with the typical placental metabolism. Notably, the bone bed of *Eremotherium* at Tanque Loma in southwest Ecuador was found to contain multiple individuals that died in a stagnant marsh or swamp. The assemblage was interpreted as evidence of herding behavior in megatheres (Lindsey et al. 2020). It was further suggested that *Eremotherium* engaged in wallowing behavior for thermoregulatory purposes akin to that observed in a modern hippopotamus (Stears et al. 2018). The modeled variation in both the density and depth of fur across the geographic range of *Eremotherium* is arguably more broadly correlated with a similar thermoregulatory strategy. Large migratory ungulates such as the American bison (*Bison bison*) shed their dense winter coat to a lighter, patchy coat that coincides with wallowing behavior (McMillan et al. 2000). Large *Megatherium* also could have engaged in wallowing but would not have required shedding due to its year-round thermal neutrality when having a dense, deep 30 mm fur coat (Figs. 5 and 6).

Social behavior dynamics of the extinct megatheres and large mylodonts (e.g., *Lestodon*) are further proposed to be consistent with those observed in large extant placental mammals (Tomassini et al. 2020; Lindsey et al. 2020), yet differ greatly from the solitary lifestyles of extant tree sloths (Taube et al. 1999). One advantage to more complex social behavior could have been shared parental care (also inferred from the bonebed of *Eremotherium* at Tonque Loma; Lindsey et al. 2020) with multiple adults protecting juveniles that are most susceptible to predation. Conversely, a sole female *Bradypus* spp. raises her single offspring, and if it falls from the tree during lactation/weaning, then she does not invest the metabolic energy to retrieve it (Taube et al. 1999).

Again, the present findings predict densely furred integument for *Mylodon* and *Nothrotheriops* as demonstrated by their mummified pelts (Ridgewood 1901; Hausman 1929). Given that there is a lack of fossil bonebeds consisting of multiple individuals, it is suggested that both genera potentially lived solitary lifestyles similar to extant tree sloths and armadillos (Naples et al. 1990). Mylodontids also possess robust limb bones that were resistant to bending forces akin to extant armadillos (Copploe et al. 2015), suggesting that some genera were capable of digging their own dens or burrows (Bargo et al. 2000). Solitary denning behavior as seen in modern brown bears (González-Bernardo et al. 2020), a body size analog of both smaller ground sloth taxa, also may have been typical of *Mylodon* and *Nothrotheriops* in cave dwellings. Moreover, an ecological preference that could have overlapped with ursids is diet. A modern species of two-toed sloth, *Choloepus hoffmanni*, is a close relative to *Mylodon* (Preslee etc. al. 2019; Delsuc et al. 2019; Tejada et al. 2024), and has been known to occasionally consume meat in captivity (Hayssen 2011*).* Species of the genus *Choloepus* are also more active compared to *Bradypus variegatus* (Sunquist and Montgomery 1973), which specializes in a diet of leaves presumably akin to its megathere relatives.

Last, while it is tempting to focus on phylogenetic constraint as the singular reason for the predicted low basal metabolic rates of extinct ground sloths, it should be noted that traits can be secondarily lost or acquired throughout the evolutionary history of a clade. Prior to GABI, the very large herbivore guild of South America was dominated by notoungulates and astrapotheres that were mixed grazers and browsers that went extinct by the end of the Miocene prior to the earliest occurrences of the exceptionally large megatherines such as *Eremotherium* and *Megatherium* (Croft et al. 2020). The largest of the notoungulates were members of the families Toxodontidae, Leontiniidae, and Homalodotheriidae. While toxodontids and macraucheniids lived alongside megatherines through the late Pleistocene (and reached body masses of 1,000–1,200 kg; Croft et al. 2020; Nelson et al. 2023), astraoptheres, leontinids, and homalodotheres ranging in body masses from 375 to 3,000 kg (Croft et al. 2020; Nelson et al. 2023) went extinct by the end of the middle to late Miocene. By contrast, most South American Miocene ground sloth genera were much smaller in size compared to contemporary notoungulates and astrapotheres, and larger ground sloths that would evolve in the Pliocene and Pleistocene (6 − 1,025 kg; Pant et al. 2014: table s1). A lower metabolic rate in ground sloths would have reduced resource demands and potentially limited competition with notoungulates and astrapotheres. This in turn could be understood as an “ecological steppingstone” towards occupying the vacant browser niche without requiring as many calories as a similarly-sized mammal with a typical placental metabolism. The observed trends in daily food intake in *Eremotherium* and *Megatherium* support this notion by suggesting little change in the amount of food consumed across their various geographic regions (Fig. 7). This major ecological factor also could have limited intraspecific competition for resources in megatheres, but notably reduced the pressure of interspecific competition with herds of proboscideans (e.g., mammoths and mastodons) with much greater energetic demands that entered South America following GABI.

### Limitations

While use of REE index values in the present study produced values within acceptable limits as reported by MacFadden et al. (2010), there are inconsistencies between this method and the phosphate δ^18^O data. Namely, FMNH P14511 has an abnormally high REE index of 36.6 and acceptable phosphate δ^18^O measurements within the range of extant placental mammals. The REE index is the first technique used to assess the degree of diagenesis in fossil xenarthran teeth, and this method has yet to be tested on specimens outside of the Pliocene and Pleistocene localities of Florida. The high REE concentration in the inner orthodentine of FMNH P14511 could be explained by it being found in older Miocene deposits, although this is inconsistent with the phosphate δ^18^O measurements which indicate that it is well-preserved. Moreover, a specimen of *Bradypus* (UF 25980) reported in the supplementary materials of MacFadden et al. (2010) has a dentine/bone index value of 30.6, which is comparable to the index value of FMNH P14511. That said, the criteria set by MacFadden et al. (2010) of acceptable REE index values > 0.35 are based on measurements of enamel-bearing mammals from the same localities as fossil xenarthrans rather than from modern bone, dentine, and enamel. We were unable to procure enamel samples from non-xenarthran placental mammals from the same localities, which would have aided in determining the relative degree of diagenesis in the ground sloth teeth used in this study. Clumped isotope temperatures are also based on CO_3_ being substituted into the PO_4_ and OH sites of hydroxylapatite, while REE concentrations occur on the Ca^2+^ site of hydroxylapatite (Kohn et al. 1999), suggesting that geochemical alteration may not be the same at either bonding site. Based on these factors, and the REE index not accounting for the carbonate contamination of UF 312,730 (Fig. 1c; Online Resource 2), we argue that use of the REE index alone does not always provide a robust indicator of diagenesis or lack thereof.

Suárez and Passey (2014) showed that clumped isotope temperatures found in fossil bone can be intermediate between ambient temperatures during the time of fossilization and body temperature. However, predominantly bone was used in their analysis, which is more prone to diagenetic alteration compared to dental tissues such as enamel and dentine (MacFadden et al. 2010). Their dataset also did not include internal orthodentine from fossil xenarthrans, which is less prone to diagenesis than external dentine but not as resistant as enamel. Cathodoluminescence performed by Larmon et al. (2019) demonstrated that internal orthodentine in an *Eremotherium* tooth exhibits very few cracks where mineral impregnation can occur and they concluded that little diagenesis had occurred. Moreover, Suárez and Passey (2014) did not compare phosphate concentrations of their samples compared to extant mammalian bone, whereas our previous studies (Eagle et al. 2010, 2011) have shown that the temperatures obtained from the teeth of extinct vertebrates using clumped isotope mass spectrometry reflect the core body temperature of the organism, and not an intermediate between the body temperature and the temperature of the environment.

The novel data generated herein offer several intriguing implications for ground sloth integument, thermal energetics, ecology, and behavior. However, it is acknowledged that the results may not be definitive. The presence of low body temperatures determined by clumped isotope paleothermometry is suggestive that ground sloths were heterothermic, akin to their closest living relatives. However, further analyses with a larger sample size consisting of specimens from various depositional environments are needed to verify this hypothesis. Given that some of the cortical bone samples did not come from the same individuals as the respective tooth samples, this could impact the values of REE ratios due to potential differences in REE uptake during fossilization. While samples were taken from other sections of the fossil teeth used for the geochemical analyses, these were not used due to restrictions of time and funding for this study. Further analyses of these samples could better indicate evidence of a past heterothermic condition as the inner orthodentine could have recorded different isotopic signals, and thus different body temperature estimates.

The modeling simulations presented here assumed consistent fur depth and density across the entire body, whereas these parameters can vary about the body surface area and this factor should be taken into account when considering the full implications of the results. Other factors such as skin thickness, hair shaft thickness, underfur, and the presence/absence of hollow hair shafts should additionally be taken into account with future analyses. Admittedly, we tested only average adult body masses, and our results could vary if tested while accounting for intraspecific variations in body masses. Pliocene climates were excluded from this analysis due to Pleistocene climate variables being easier to use for extrapolation of reasonable monthly values from modern climate data. The inclusion of Pliocene Epoch climate data would give more insight into how integument and associated fur coverage could have varied in earlier populations of both *Eremotherium* and *Megatherium*. Lastly, geologic ages of localities sampled for the microclimate simulations cover a broad range of geologic time encompassing the entire span of the Pleistocene from 2,500,000–11,500 years ago, and more precise dates were not available in the Paleobiology Database (Online Resource 1). More precise estimates of paleoclimate and dating of ground sloth localities could have greatly improved the microclimate simulations.

## Conclusions

Clumped isotope palaeothermometry of inner orthodentine provides convincing evidence for extinct ground sloths having body temperatures akin to extant xenarthrans and potentially being heterothermic, regardless of absolute differences in body sizes. These findings combined with novel biophysical modeling results in Niche Mapper demonstrate that lower body temperatures and basal metabolisms, in addition to environmental variables, impacted ground sloth geographical distribution, thermoregulation ability, and integument form. The “hairless model of integument” previously proposed for *Megatherium* is untenable when more accurate estimates of xenarthran basal metabolism, while the closely related *Eremotherium* has been suggested to be sparsely furred in neotropical climates. Giant ground sloths likely had a variety of integument appearances from dense, thinner fur coverage in the neotropics to dense, thicker fur coverage in cold environments. Smaller ground sloths would have needed dense, thick fur supplemented with behavioral thermoregulation to endure cold climates throughout the year. The modeling data also point to surprising adaptability of xenarthran physiology in both the largest and smaller ground sloths. Moreover, the predicted integument models for the largest taxa are believed to be reliable unless refuted by a future specimen with extensive preserved integument. Prospective future models must examine other taxa (e.g., *Megalonyx*, *Thalassocnus*, *Lestodon*, etc.) and include histological studies to help determine how low the basal metabolic rates of ground sloths could have been beneficial, therefore providing further insight into the physiology of these and other fossil xenarthrans and how their metabolic rates impacted their integument appearance, growth rates, ecology, and behavior.

## Electronic supplementary material

Below is the link to the electronic supplementary material.

Supplementary Material 1

Supplementary Material 2

## Data Availability

All data collected and analyzed for this study are included in this published article in adherence with disclosure policy of the journal. The authors are sharing new data alongside the results of this report; Niche Mapper input has been reposited to Zendo (10.5281/zendo.10626431) and clumped isotope data has been reposited to EarthChem (10.60520/IEDA/113127).
